# Whole-Brain Mapping of Neuronal Activity Associated with Vocal Socialization Behaviors in Adult Mice

**DOI:** 10.1523/ENEURO.0400-25.2026

**Published:** 2026-05-14

**Authors:** Shi-Xiang Luo, Shih-Yun Chen, Hsiao-Ying Kuo, Fu-Chin Liu

**Affiliations:** ^1^Institute of Neuroscience, National Yang Ming Chiao Tung University, Taipei 112304, Taiwan; ^2^Institute of Anatomy and Cell Biology, National Yang Ming Chiao Tung University, Taipei 112304, Taiwan

**Keywords:** immediate early gene, medial prefrontal cortex, social communication, striosome, ultrasonic vocalizations

## Abstract

Vocal communication is essential for social behavior, yet the distributed brain networks underlying vocal production remain elusive. Male mice produce ultrasonic vocalizations (USVs) during courtship, providing a rodent model for investigating neural circuits underlying innate vocal communication. Here, we used a double-labeling strategy combining genetic activity tagging (TRAP2) and c-Fos immunohistochemistry to generate an unbiased, whole-brain map of neuronal ensembles activated during courtship-induced USV production in adult male mice. By tracking neuronal activity across 25 brain regions during two independent courtship sessions, we identified populations consistently recruited during social vocalization. Quantitative analyses revealed robust activation in the caudal periaqueductal gray, confirming its established role as a hub for vocal motor control. Importantly, correlation analyses between neuronal activity and USV count distinguished regions specifically linked to vocal output from those associated with general social interaction. The medial prefrontal cortex, particularly the prelimbic and cingulate cortices, exhibited strong positive correlations with USV production, suggesting an integrative role in translating the social and motivational context into vocal commands. Notably, the rostral striatum showed a strong correlation with USV count, with vocalization-related activity preferentially localized within striosome compartments of the medial striatum. Given that striosomes receive limbic cortical input and are implicated in motivational processing, these findings suggest that corticostriatal limbic–motor circuits modulate innate vocalization contingent on emotional and social contexts. Collectively, our study proposes a comprehensive neuroanatomical framework linking cortical motivation centers, striatal limbic–motor pathways, and brainstem vocal motor circuits, providing insights into the distributed neural architecture underlying mammalian social communication.

## Significance Statement

Mouse ultrasonic vocalizations (USVs) provide a mammalian model for investigating the neural circuits underlying social communication. Using whole-brain activity mapping with double labeling of TRAP2 and c-Fos, we identified a distributed network extending from the medial prefrontal cortex and striatum to the periaqueductal gray that is recruited during courtship-induced vocalization. The discovery that USV-related neurons in the striatum are preferentially localized to striosomal compartments reveals a potential limbic–motor interface for integrating social motivation with vocal motor output. Together, these findings identify a multilevel neural network that may be evolutionarily conserved for integrating motivation and motor control in mammalian communication.

## Introduction

Rodents use ultrasonic vocalizations (USVs) as a key mode of social communication across a variety of contexts, including maternal care, aggression, and courtship (Holy and Guo, 2005; Arriaga et al., 2012). In adult male mice, USVs are produced during courtship encounters with females and are thought to play an important role in mate attraction and reproductive success (Asaba et al., 2017). Due to their social relevance, USVs have been widely employed as a behavioral model to study the neural mechanisms underlying mammalian vocal communication.

Previous studies in neuroethology and systems neuroscience have established the brainstem periaqueductal gray (PAG) as a conserved hub for vocal readiness across vertebrate species and that motor neurons located in the nucleus ambiguus coordinate respiration and execute vocalization (Jürgens, 2009; Arriaga et al., 2012; Veerakumar et al., 2023; Park et al., 2024). In mice, lesions or optogenetic silencing of caudal PAG neurons abolish USV production, whereas stimulation can elicit vocal output (Tschida et al., 2019; Michael et al., 2020). These findings suggest that the PAG is necessary for producing innate USVs. However, the PAG does not act in isolation. It receives diverse inputs from cortical, limbic, and hypothalamic structures, suggesting that higher-order circuits contribute to shaping the initiation, motivation, and context dependence of vocal behavior (Jürgens, 2009; Tschida et al., 2019; Michael et al., 2020). In parallel, the nucleus ambiguus receives descending forebrain inputs conveying integrative information related to learned vocalizations, thereby modulating vocal motor output, particularly in vocal learners (Jürgens, 2009; Arriaga et al., 2012; Nieder and Mooney, 2020).

Increasing evidence indicates that the medial prefrontal cortex (mPFC) and basal ganglia are involved in vocalization. The mPFC integrates motivational and social cues (Grossmann, 2025) and projects to subcortical vocal centers, including the PAG (Jürgens, 2002). Recent studies have shown that stimulation of neurons in the anterior cingulate cortex and prelimbic cortex can trigger USV production in rodents (Bennett et al., 2019; Gan-Or and London, 2023), suggesting the causal role of prefrontal circuits. Likewise, corticostriatal pathways, particularly striatal striosomes, have been implicated in affective and motivational processing (Crittenden and Graybiel, 2011). Although the corticostriatal pathways are best known for their roles in learned vocal behaviors in songbirds and humans (Scharff and Nottebohm, 1991; Jarvis, 2004, 2019; Aronov et al., 2008; Jürgens, 2009), their contribution to innate mouse USVs remains elusive.

The dorsal striatum is organized into two neurochemically distinct compartments, the labyrinthine striosomes and the surrounding matrix (Graybiel and Ragsdale, 1978; Kumar, 2012). This mosaic architecture is fundamentally defined by differential connectivity, where striosomes serve as the primary interface with the limbic system. Unlike the matrix, which predominantly processes sensorimotor information, striosomes receive preferential inputs from limbic cortices, including the anterior cingulate and orbitofrontal cortex (Crittenden and Graybiel, 2011; McGregor et al., 2019). Uniquely, striosomal projection neurons send outputs directly to dopamine-containing neurons in the substantia nigra pars compacta and indirectly to the lateral habenula (LHb) via the pallidum, forming limbic-associated loops (Crittenden et al., 2016; Hong et al., 2019; Matsushima and Graybiel, 2020). These limbic-associated circuits allow striosomes to integrate motivational states with cognitive control, playing a critical role in reinforcement learning and value-based decision-making under conflict (Friedman et al., 2015; Bloem et al., 2017, 2022). Despite the role of striosomes in valuation and reward prediction, it is yet unclear whether striosomes participate in socially motivated vocalization.

Despite these advances, the broader network-level architecture that supports social vocalization in mice has not been systematically mapped. Prior studies often focused on individual regions or circuits, leaving open questions about how cortical, striatal, and brainstem structures are recruited together during naturalistic social interactions. Immediate early gene (IEG)-based activity mapping offers an unbiased strategy for identifying neuronal ensembles engaged during complex behaviors (Guenthner et al., 2013; Allen et al., 2017; DeNardo et al., 2019). Applying such approaches to USV production may reveal both canonical vocal control centers and novel contributors to social communication.

In the present study, we employed a double-labeling strategy combining genetic activity tagging and c-Fos immunohistochemistry to generate a whole-brain map of neuronal ensembles recruited during courtship-induced USV production in male mice. Through correlation analyses, we attempted to distinguish regions broadly engaged by social interaction from those specifically linked to vocal output. We identified a distributed network extending from the mPFC and striatum to the PAG and highlighting candidate circuits for integrating motivational state with vocal motor control. By delineating the neuroanatomical framework of mouse social vocalization, our study provides new insights into the neural circuits potentially involved in the interaction between social motivation and vocalization.

## Materials and Methods

### Animals

*Fos[2A-iCreER]* (TRAP2) mice (Jackson Laboratory #030323) were used as the primary strategy for capturing activated neurons. For whole-brain mapping experiments, *TRAP2* mice were intercrossed with *Sor[tm14(CAG-tdTomato)Hze]* (Ai14; Jackson Laboratory #007914) mice to generate *TRAP2;Ai14* double-transgenic mice. Female wild-type (WT) mice (C57BL/6JNarl; ∼12 weeks old) served as mating partners during behavioral tasks and were either purchased from the National Center for Biomodels or bred in-house. All mice were housed on a 12:12 h light/dark cycle and were single-housed for at least 1 week prior to experimental procedures. All surgical and behavioral procedures were approved by the IACUC of National Yang Ming Chiao Tung University.

To gain systematic insight into brain regions involved in USV production in mice, we tracked neuronal activity across the entire brain of male mice during courtship behavior. Rather than using electrophysiology or fiber photometry, which are restricted to monitoring small brain portions, we employed the IEG c-Fos as a marker of neural activation. Following courtship behavior and USV emission, c-Fos is expressed in activated neurons and reaches peak expression 90–120 min poststimulation (Numan and Numan, 1994). This transient expression window makes c-Fos an ideal marker for time-locked neuronal activation. By mapping c-Fos expression across brain regions in vocalizing mice, we attempted to identify candidate areas implicated in USV production.

### Drug administration

**Figure 1.**
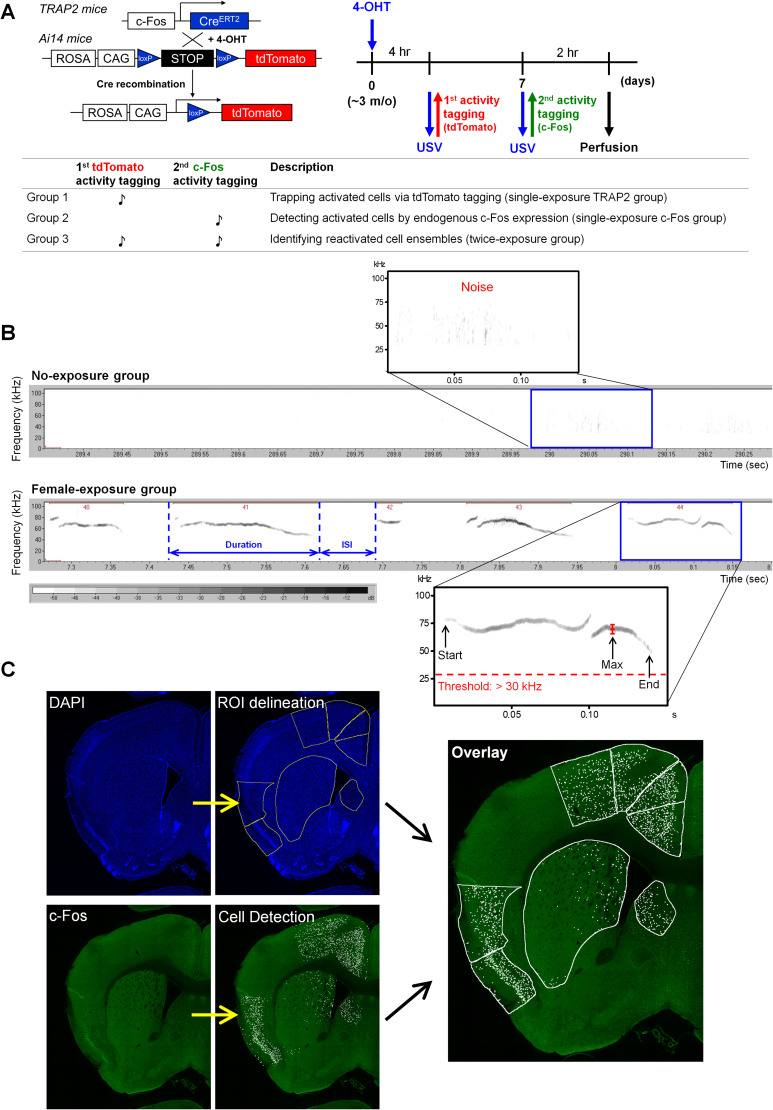
Experimental workflow for activity tagging, USV analysis, and c-Fos quantification. ***A***, Schematic experimental design. *TRAP2* mice were crossed with *Ai14* reporter mice. Following 4-OHT administration, neurons activated during the first female exposure were permanently tagged with tdTomato mediated by Cre recombination. A second female exposure was conducted 7 d later to induce endogenous c-Fos expression for detecting activated neurons. Group allocation for reactivation analysis is summarized in the table. ***B***, Representative USV spectrograms from the no-exposure (top) and female-exposure (bottom) groups. The no-exposure group showed no detectable USV signals, except for low-intensity background noise (top). In contrast, USV syllables (>30 kHz) emitted by the female-exposure group were automatically detected, with intersyllable intervals (ISIs) longer than 20 ms defining separated events. Color intensity indicates relative amplitude in decibels (dB). ***C***, Automated workflow for ROI delineation and activated cells mapping. ROIs were delineated on DAPI-stained sections with ImageJ software (top), and activated cells were detected with the QuPath software (bottom). The identified cells were subsequently quantified separately according to each ROI (right).

To tag neurons activated during USV emission, *TRAP2* mice received intraperitoneal injection of 4-hydroxytamoxifen (4-OHT; 50 mg/kg in 5% Tween 80) 4 h prior to female interaction at Postnatal Day (P)90. Four hours prior to the initial behavioral task, we administered 4-OHT intraperitoneally to *TRAP2;Ai14* mice. Upon neuronal activation, Cre recombinase is coexpressed with c-Fos. Once bound to 4-OHT, Cre recombinase translocates to the nucleus and excises the loxP-flanked STOP cassette. This recombination enables activated neurons to express tdTomato, allowing fluorescence microscopy visualization. Following the first labeling, the mice rested for 7 d to ensure that c-Fos expression returned to baseline and sufficient tdTomato accumulated in the labeled neurons. On day eight, mice underwent the same courtship behavioral task, with activated neurons visualized using c-Fos immunostaining. Mice were perfused 2 h postbehavioral task when c-Fos expression peaked. USVs were recorded throughout both behavioral sessions for subsequent correlation analysis with neural activation patterns (Fig. 1).

### USV recording and analysis

All recording sessions were conducted after male mice reached P90. To elicit USVs for activity tagging, single-housed males were placed in a soundproof recording chamber for each acquisition session. After a 10 min habituation phase, a novel free-moving female mouse was introduced into the chamber for an additional 5 min. The chamber was kept empty, with no food or water provided, to exclude feeding or drinking behaviors. Throughout the entire session, USVs were recorded using an ultrasonic microphone (Avisoft Bioacoustics CM16) positioned 15 cm above the home cage. Simultaneously, signals were digitized using a recording system (Avisoft UltraSoundGate 116) and Avisoft-RECORDER software. For USV detection, raw audio data were first filtered to remove common noise sources (cutoff frequency, 30–120 kHz) and then segmented using a spectral bandwidth algorithm (50% of total energy; hold time, 20 ms; postfilter, 1 ms). The following USV features were extracted for subsequent analysis: number of events, event duration, start/end time, peak frequency, and peak amplitude at three different locations within each event (start of event, end of event, and maximum amplitude of event). In the present study, we focused on the analysis of call events. We continuously monitored all behavioral sessions using video recording and confirmed the absence of any distress-related behaviors.

### Brain tissue preparation

Mice were anesthetized with isoflurane and transcardially perfused with 0.9% saline, followed by 4% paraformaldehyde (PFA) in 0.1 M phosphate-buffered saline (PBS), pH 7.4. Perfused brains were postfixed in 4% PFA at 4°C overnight and subsequently cryoprotected with 30% sucrose in 0.1 M PBS for 48 h. Brain tissues spanning coordinates from the bregma +2.9 to −5.0 mm were sectioned into 30-μm-thick coronal sections using a cryostat (Leica).

### Immunohistochemistry

Brains were sectioned into 30 μm free-floating coronal slices. Sections were collected sequentially into 12 parallel series (intersection interval of 360 μm within each series). One representative series was processed for immunostaining to ensure systematic sampling across the entire rostrocaudal axis. The coronal sections were washed three times with 0.1 M PBS (3 × 5 min) and permeabilized for 15 min at room temperature with 0.2% Triton X-100 in 0.1 M PBS (PBST). Endogenous peroxidase activity was quenched by incubating sections with 3% H_2_O_2_ and 10% methanol for 5 min. Sections were then incubated for 1 h with 3% normal donkey serum (NDS; Sigma-Aldrich, G6767) in PBS at room temperature. Following blocking, sections were incubated in a primary antibody cocktail (0.1 M PBS containing 1% NDS, 0.2% Triton X-100, 0.1% sodium azide, and optimal concentration of primary antibody) at 4°C for at least 48 h. The primary antibodies were rabbit anti-c-Fos (1:500, Merck, #ABE457; 1:1,000, ABclonal Technology; #A2444), rabbit anti-MOR1 (1:1,000, ImmunoStar, #24216) and guinea pig anti-MOR1 (1:500, Merck; #AB5509). For c-Fos immunofluorescence, sections were incubated with fluorophore-conjugated secondary antibodies (1:500) in 0.1 M PBS for 1 h following primary antibody binding. Sections were then counterstained with 4′,6-diamidino-2-phenylindole (DAPI; 1:5,000) for 10 min. For MOR1 immunofluorescence, sections were incubated with biotinylated secondary antibodies (1:500) in 0.1 M PBS for 1 h following primary antibody binding, then with avidin–biotin–peroxidase complex (1:200, PK-6100, Vector Laboratories). Tyramide signal amplification reagent (1:2,000, PerkinElmer) was subsequently applied to complete the staining process. Sections were counterstained with DAPI.

### Image processing and cell counting

Whole-brain mapping images were acquired using the Olympus BX63 and Zeiss Axioscan 7 microscopes. Images with DAPI staining were first processed using ImageJ software. Twenty-five brain regions distributed across different anatomical locations were manually delineated, referencing the Atlas of the Adult Mouse Brain and previously described criteria (Van De Werd et al., 2010) by Researcher 1, and then confirmed by Researcher 2. Subsequently, tdTomato/c-Fos fluorescent images with brain region annotations were analyzed using the open-source bioimage analysis software, QuPath (Bankhead et al., 2017), for automated quantification. This approach was adopted to eliminate potential human error and subjective bias associated with manual counting. Cell signals were detected using the QuPath algorithm by adjusting parameters, including nucleus background radius, median filter radius, and intensity threshold. For tdTomato signal detection, the background radius was set to 10 μm, the median filter radius to 10 μm, and the intensity threshold to 20. For c-Fos signal detection, the background radius was set to 10 μm, the median filter radius was set to 8 μm, and the intensity threshold was set to 10. To validate accuracy and consistency, cell detection was performed using both QuPath and manual counting. Detection parameters and thresholds were adjusted until the variability between the two approaches was <15%, with no statistical differences observed. When QuPath detection results required refinement, the detection algorithm was further improved using QuPath's integrated AI tools. QuPath quantified not only cell numbers but also provided additional measurements, including fluorescent intensity, area of interest, and double-labeled cell counts, enabling detailed comparative analyses. Cell counts were normalized to the counted area and expressed as density (number of positive cells/square millimeter). The number of double-labeled cells was divided by the total number of tdTomato^+^ or c-Fos^+^ cells to obtain the percentage of double labeling. For each mouse, signal density was averaged across all sections within each region of interest (ROI).

### Statistical analysis

All data were tested for normality prior to statistical analysis. For datasets following a normal distribution, either independent or paired two-tailed *t* tests were used, depending on experimental design. When comparisons involved multiple groups or factors, two-way analysis of variance (ANOVA) was conducted, followed by Tukey's honestly significant difference post hoc test to adjust for multiple comparisons. These data are presented as mean ± standard error of the mean. For datasets that did not meet the assumption of normality, nonparametric tests were applied. Specifically, the Mann–Whitney *U* test was used for between-group comparisons, while the Wilcoxon signed-rank test was applied for within-subject comparisons. These data are presented as median ± interquartile range. All statistical analyses were performed using the SPSS software, with the threshold for statistical significance set at *p* < 0.05. The *z* score for each brain region was calculated using the following formula: *z* = (*x* − *μ*)/*σ*, where *μ* and *σ* represent the mean and standard deviation, respectively, of the number of c-Fos^+^ cells across seven animals and *x* is the raw cell count for a given region. Positive and negative *z* scores indicate relatively high and low c-Fos expression, respectively. Pearson's correlation coefficient (*r*) was used to assess the relationship between the number of USVs and c-Fos^+^ cell counts. All statistical data, test details, and 95% confidence interval values are summarized in the statistical table (Extended Data Table 2-1). All sample sizes (*n*) for all statistical comparisons are reported in their corresponding figures or figure legends.

10.1523/ENEURO.0400-25.2026.t2-1 Table 2-1 Statistical Table. Download Table 2-1, DOCX file.

## Results

### Unbiased whole-brain mapping of courtship USV-related neurons in mice

Previous studies have demonstrated that c-Fos immunostaining alone provides sufficient resolution to draw preliminary conclusions about neuronal activation (Kim et al., 2015; Mai et al., 2023; Hu et al., 2024). However, since neuronal activity occurs continuously throughout the brain, with variable basal activity levels between individuals, these variations can introduce noise or bias in the interpretation of data. To minimize the impact of individual differences, we developed a double-labeling approach wherein each mouse underwent the same courtship behavior task twice (Fig. 1*A*). Neurons activated during each session were labeled using distinct methods, generating two independent signal sets corresponding to neurons activated during courtship and vocalization behaviors. By analyzing the overlap between these signals, we identified double-labeled neurons that could be linked to courtship behavior with greater confidence, as they were consistently activated across independent trials. This approach required a system enabling repeated labeling of activated neurons in living subjects without interference between labeling events (Fig. 1*B*; also see Materials and Methods). We satisfied this requirement by combining IEG properties with the Cre-loxP system using two transgenic mouse strains: *TRAP2* and *Ai14*. In the *TRAP2* line, a tamoxifen-inducible, improved Cre recombinase (iCreERT2) construct is placed under *Fos* promoter control (Guenthner et al., 2013; Allen et al., 2017). In the *Ai14* line, a loxP-flanked STOP cassette prevents tdTomato reporter gene transcription. Crossing these lines generated *TRAP2;Ai14* transgenic mice (Fig. 1*A*; also see Materials and Methods).

### Identification of courtship-related cell ensembles in the mouse brain

For quantitative analysis, we selected 25 brain regions spanning the forebrain to the midbrain based on a previous transsynaptic tracing study. This prior study demonstrated that these areas provide direct input to PAG–USV neurons, whose activity is both necessary and sufficient for social vocalization production in mice (Michael et al., 2020). Brain regions were manually segmented based on DAPI staining (Fig. 1*C*), referencing the Adult Mouse Brain Atlas and established cytoarchitectonic studies (Sah et al., 2003; Van De Werd et al., 2010; Vogt and Paxinos, 2014). By integrating anatomical mapping with quantitative imaging (Fig. 1*C*), we identified brain regions exhibiting significantly elevated neuronal activation during courtship, providing insights into their potential vocalization circuitry.

Following c-Fos immunostaining, we detected two fluorescent signal sets, tdTomato and c-Fos, within the same brain sections (Fig. 1*A*). We analyzed three datasets: tdTomato-only (Group 1; single-exposure TRAP2 group), c-Fos-only (Group 2; single-exposure c-Fos group), and double-labeled signals (Group 3; double-exposure group). Control groups received identical treatments but lacked female mouse exposure during behavioral testing sessions (no-exposure group). Since behaviorally relevant neurons may cluster within functionally specialized subregions, analyzing large regions as unified entities may overlook important activation patterns. Therefore, we subdivided large brain structures spanning broad rostrocaudal extents into anatomically defined subregions, including caudoputamen (CPu) and PAG. The CPu was divided into rostral, caudal, and tail compartments using +0.62 and −1.02 mm from the bregma as anatomical boundaries (Hunnicutt et al., 2016; Miyamoto et al., 2019; Ogata et al., 2022). Similarly, the PAG was divided into rostral and caudal subregions, with −4.2 mm from the bregma as the division point (Tschida et al., 2019).

**Figure 2.**
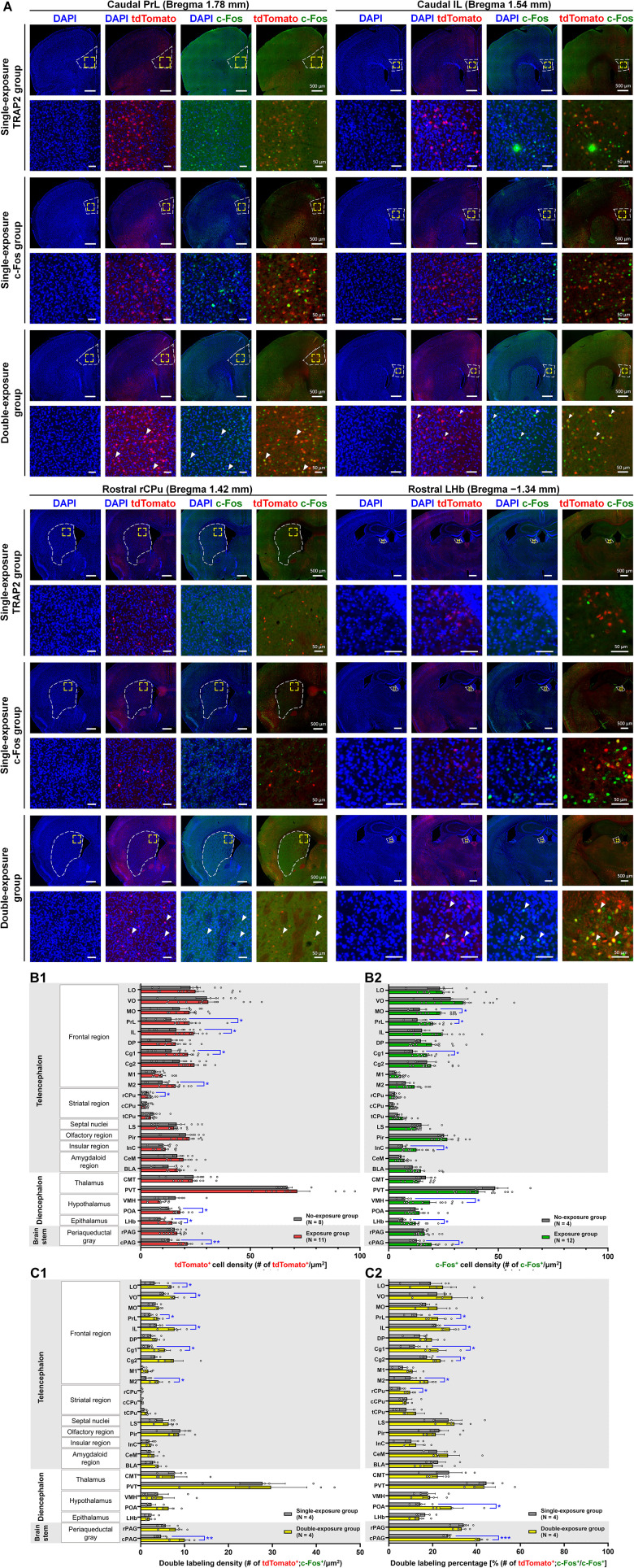
Whole-brain mapping of vocalization ensembles with an activity-tagging strategy. ***A***, Representative immunofluorescence images of tdTomato^+^ and c-Fos^+^ cells in the PrL, infralimbic (IL), cingulate cortex (Cg1), secondary motor cortex (M2), rCPu, POA, LHb, and caudal periaqueductal gray (cPAG) at rostral and caudal levels. Activated cells during a single female exposure were identified by tdTomato expression and c-Fos immunoreactivity in the single-exposure tdTomato and c-Fos groups, respectively. In the double-exposure group, colocalization of tdTomato and c-Fos signals (arrowheads) indicates reactivated neurons. Dashed boxes delineate the quantified ROI for each nucleus. Boxed regions are shown at higher magnification below. ***B***, Densities of tdTomato^+^ (B1) and c-Fos^+^ (B2) cells across brain regions following female exposure compared with the no-exposure group. ***C***, Numbers of double-labeled reactivated cells were normalized to the area of ROI (C1) and to the total c-Fos^+^ cells within each ROI (C2) across brain regions. *p < 0.05; **p < 0.01; ***p < 0.001.

Analysis of the tdTomato group revealed significantly increased tdTomato-labeled cell density in several brain regions compared with controls, including the prelimbic cortex (PrL; 13.957 ± 2.299 vs 22.321 ± 2.593; *p* < 0.05), infralimbic cortex (IL; 16.224 ± 3.221 vs 24.317 ± 2.126; *p* < 0.05), cingulate cortex area 1 (Cg1; 14.103 ± 2.792 vs 21.551 ± 2.014; *p* < 0.05), secondary motor cortex (M2; 10.001 ± 2.053 vs 15.962 ± 1.802; *p* < 0.05), rostral CPu (rCPu; 2.812 ± 0.638 vs 4.665 ± 0.543; *p* < 0.05), preoptic area (POA; 12.898 ± 1.384 vs 18.075 ± 1.105; *p* < 0.05), LHb nucleus (9.037 ± 3.434 vs 14.446 ± 3.592; *p* < 0.01), and caudolateral PAG (cPAG; 13.140 ± 1.116 vs 21.337 ± 1.345; *p* < 0.01; Fig. 2*A*,*B*1; Extended Data Fig. 2-1; Extended Data Table 2-1). These elevations suggest potential involvement of these regions during the behavioral task.

10.1523/ENEURO.0400-25.2026.f2-1 Figure 2-1 Download Figure 2-1, DOCX file.

In the c-Fos induction experiment, compared with controls without female exposure, we observed significantly increased c-Fos^+^ cell density in medial orbital cortex (MO; 17.761 ± 3.319 vs 22.264 ± 2.353; *p* < 0.05), PrL (13.175 ± 2.363 vs 20.235 ± 1.270; *p* < 0.05), Cg1 (11.115 ± 1.816 vs 17.505 ± 1.299; *p* < 0.05), insular cortex (InC; 6.509 ± 0.900 vs 12.699 ± 1.554; *p* < 0.01), ventromedial hypothalamus (VMH) nucleus (7.527 ± 1.424 vs 18.800 ± 2.813; *p* < 0.01), LHb (6.710 ± 1.218 vs 12.389 ± 1.389; *p* < 0.05), and cPAG (12.820 ± 1.425 vs 19.841 ± 1.768; *p* < 0.01; Fig. 2*A*,*B*2; Extended Data Fig. 2-1; Extended Data Table 2-1). Compared with the tdTomato dataset, MO, InC, and VMH exhibited statistical significance exclusively in the c-Fos dataset. This discrepancy may reflect differences in basal activity or methodological labeling sensitivity.

Importantly, double-labeling analysis revealed significantly higher density of neurons coexpressing tdTomato and c-Fos in lateral orbital cortex (LO; 3.173 ± 1.116 vs 6.974 ± 0.565; *p* < 0.05), ventral orbital cortex (VO; 5.288 ± 0.398 vs 7.820 ± 0.711; *p* < 0.05), PrL (2.227 ± 0.559 vs 3.987 ± 0.403; *p* < 0.05), IL (3.653 ± 0.630 vs 7.710 ± 1.397; *p* < 0.05), M2 (1.362 ± 0.500 vs 4.075 ± 0.913; *p* < 0.05), and cPAG (4.646 ± 0.709 vs 9.993 ± 1.229; *p* < 0.01; Fig. 2*C*1). We further calculated the proportion of double-labeling neurons among all and c-Fos^+^ cells (Fig. 2*C*2; Extended Data Table 2-1). Analysis using total c-Fos^+^ cells as denominator revealed significant increases in PrL (12.902 ± 2.456 vs 22.439 ± 2.850; *p* < 0.05), IL (21.730 ± 0.761 vs 27.907 ± 1.907; *p* < 0.05), Cg1 (11.965 ± 2.492 vs 22.632 ± 3.578; *p* < 0.05), cingulate cortex area 2 (Cg2; 17.392 ± 1.398 vs 23.544 ± 1.909; *p* < 0.05), M2 (10.001 ± 2.625 vs 17.996 ± 1.716; *p* < 0.05), POA (14.350 ± 2.182 vs 28.619 ± 5.321; *p* < 0.05), and cPAG (27.023 ± 0.724 vs 41.580 ± 1.250; *p* < 0.001). These results indicate that these brain regions contain neuronal populations repeatedly activated during social vocalization, supporting their involvement in vocal production.

In summary, these convergent datasets indicate that a distributed network of cortical and subcortical regions is recruited during courtship, accompanied by social vocalization, with frontal cortical areas and brainstem structures exhibiting particularly robust functional activation.

### Heatmap-based analysis of vocalization-related cell ensembles

Although elevated neuronal activity in certain regions suggests potential roles in USV production, an alternative explanation for global tdTomato/c-Fos expression after courtship is that courtship behavior itself may elicit neuronal activation related to general social interaction. To address this concern, we introduced an additional criterion: if a brain region specifically participates in USV production, its c-Fos expression level should exhibit significant correlation with the number produced.

Based on relative changes in the c-Fos expression level and USV count, brain regions can be preliminarily categorized into three groups: unrelated, social interaction-related, and USV production-related. “Unrelated” regions exhibit consistently high or low c-Fos expression regardless of USV count. “Social interaction-related” regions show altered c-Fos expression independent of USV number. “USV production-related” regions display a positive or negative correlation between c-Fos expression and USV number (Mai et al., 2023).

**Figure 3.**
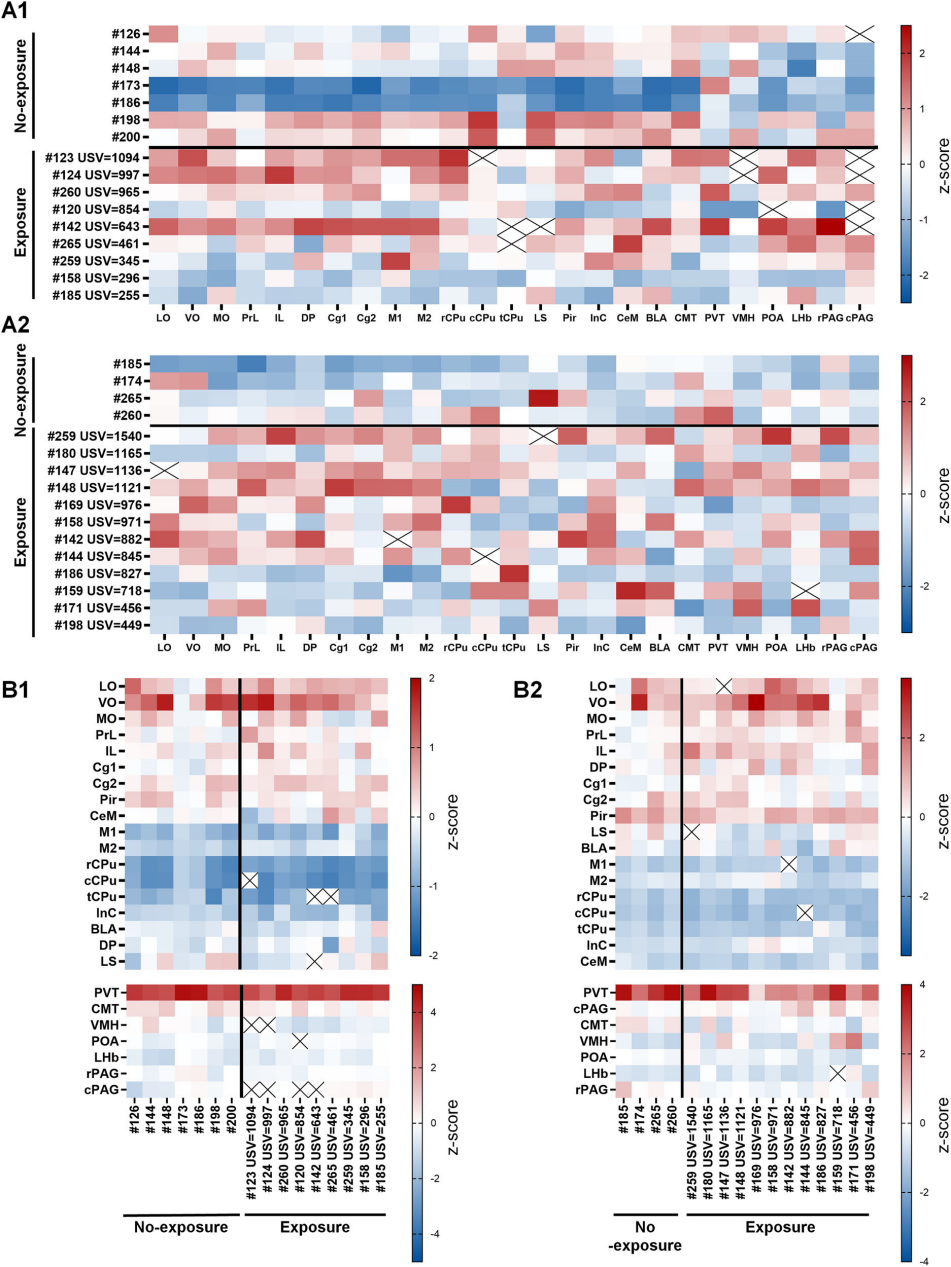
Whole-brain comparison of relative changes of cell activation following female exposure. ***A***, Heatmap showing *z* scores obtained from across-mouse normalization within each brain region in the no-exposure and female-exposure groups, using tdTomato^+^ (A1) or c-Fos^+^ (A2) cell densities from all samples within each region as a single population for normalization. Each row represents an individual mouse, and each column corresponds to a brain region. Red and blue indicate higher and lower activation, respectively, relative to the group mean. Rows are sorted according to the total number of USVs emitted by each mouse in the female-exposure group. ***B***, Heatmap showing *z* scores obtained from within-mouse normalization across regions in the no-exposure and female-exposure groups, using tdTomato^+^ (***B*1**) or c-Fos^+^ (***B*2**) cell densities from all regions within each individual mouse as a single population for normalization. Each row represents a brain region, and each column corresponds to an individual mouse. Red and blue indicate higher and lower activation, respectively, relative to the group mean. Data are sorted according to the total number of USVs emitted by each mouse in the female-exposure group. Missing values (black crosses) indicate statistical outliers excluded before *z* score conversion.

We revisualized data from Figure 2 using heatmaps. tdTomato and c-Fos signal densities were normalized using two complementary *z*-scoring approaches across mouse samples and brain regions: across-mouse normalization within each region (Fig. 3*A*1,*B*1) and within-mouse normalization across regions (Fig. 3*A*2,*B*2).

Under across-mouse normalization (Fig. 3*A*1), the female-exposure group consistently exhibited higher tdTomato signal densities in mPFC subregions (PrL, IL, Cg1, M2) and cPAG, indicated by red shading (*z* score > 0). Conversely, the non-USV group showed values near or below the population mean (blue to neutral shades). Notably, when mice were ordered by total USV count, mPFC subregions in female-exposure mice displayed a clear red-to-blue gradient, suggesting a positive correlation between USV number and activation of these brain regions. Conversely, regions such as lateral septum (LS) and VMH exhibited opposite color gradients, indicating a negative correlation between USV number and neuronal activity. These patterns suggest these regions may be “USV production-related” areas. Other regions showing activity changes compared with controls but lacking color gradients were motor cortex area 1 (M1), piriform cortex (Pir), and others, likely representing “social interaction-related” regions.

For c-Fos signals, across-mouse normalization (Fig. 3*B*1) also revealed elevated activity in most mPFC subregions; however, unlike tdTomato data, consistent gradients corresponding to USV count were not observed in female-exposure mice. Additionally, in both tdTomato and c-Fos heatmaps, subcortical structures such as POA, LHb, centromedian thalamic nucleus (CMT), and rostral PAG (rPAG) displayed more variable activation patterns across mice.

In contrast to across-mouse normalization, within-mouse normalization (Fig. 3*A*2,*B*2) emphasizes relative differences across brain regions within each individual. By controlling interanimal variability, we identified areas most strongly activated in each mouse. For tdTomato signals (Fig. 3*A*2), although mPFC subregions (VO, PrL, IL, Cg2) and cPAG showed relatively high neuronal activity (red) in both no-exposure and female-exposure groups, activation levels were generally greater in the female-exposure group. When female-exposure mice were ordered by USV counts, VO, PrL, and IL again displayed red-to-blue gradients, indicating these regions may be USV production related. Other highly activated regions, such as Pir, paraventricular thalamic nucleus (PVT), and CMT, lacked clear gradients and therefore likely represent “social interaction-related” regions. Similarly, for c-Fos signals (Fig. 3*B*2), mPFC subregions tended toward high within-animal activation in female-exposure mice, but no clear USV count gradient emerged, mirroring across-mouse normalization findings.

In summary, both normalization approaches consistently identified PrL, IL, Cg1, Cg2, and M2, as well as cPAG, as the most robustly activated regions during courtship behavior based on color gradients, suggesting potential links between USV production and neuronal activity. In contrast, other areas (e.g., POA, LHb, PVT, and CMT) may reflect broader social interaction aspects.

### Heatmap-based identification of vocalization-related cell ensembles: correlation analysis

**Figure 4.**
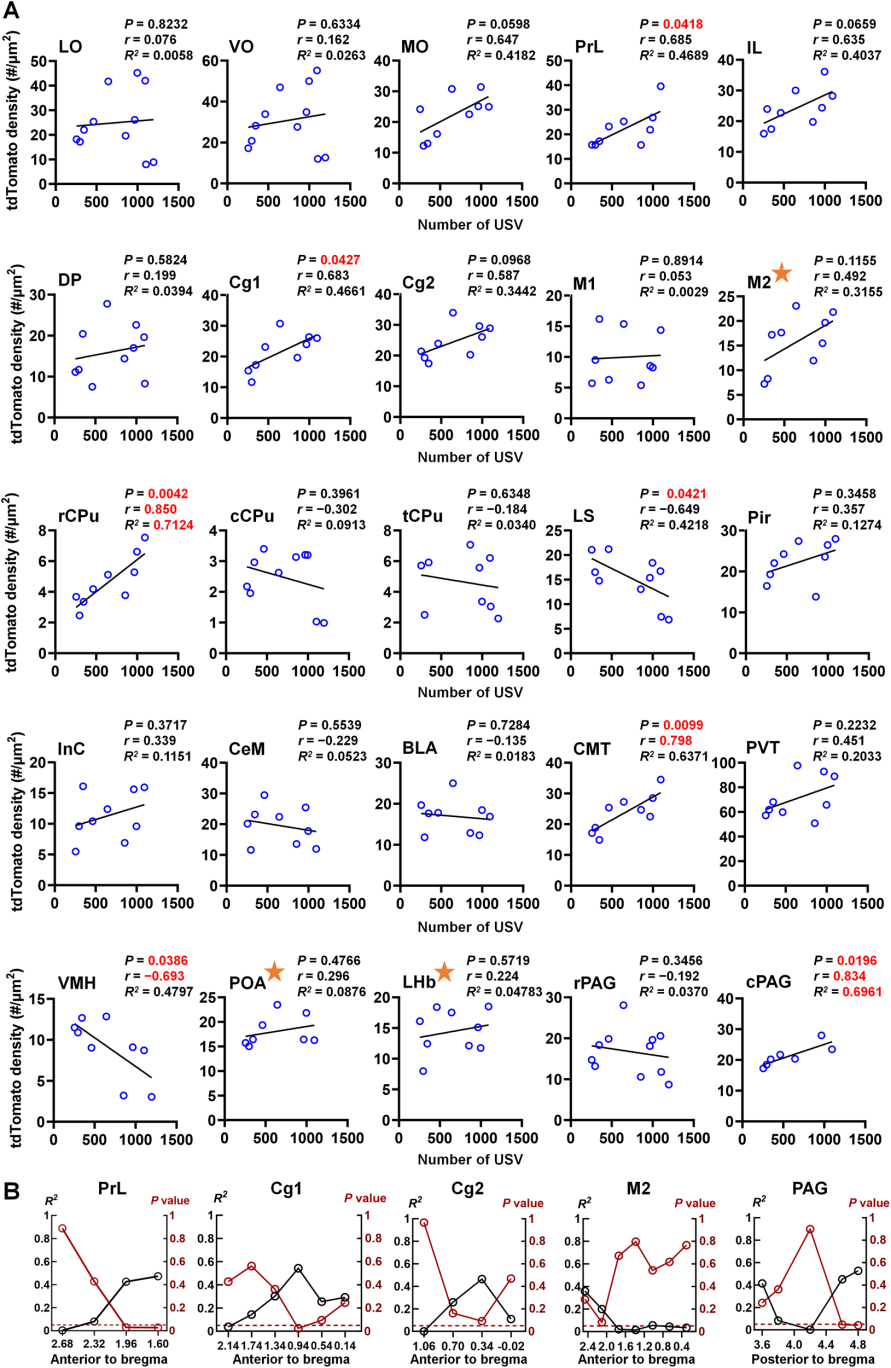
Correlation between neuronal activation and the number of USV emissions during female exposure across brain regions. ***A***, Scatterplots showing the correlations between tdTomato^+^ cell density and the number of USVs emitted during female exposure. Each panel represents an individual brain region. Linear regression lines are shown with *p* value, correlation coefficients (*r*), and coefficient of determination (*R*^2^) from Pearson's correlation. Values of *r* and *R*^2^ shown in red indicate coefficients greater than 0.7 and 0.5, respectively, reflecting a high level of correlation. Regions highlighted with orange stars indicate significantly elevated densities of tdTomato^+^ cells shown in Figure 2, but no statistically significant correlations with USV number. ***B***, Anteroposterior anatomical mapping of correlation strength (*R*^2^, black) and *p* values (red) across representative regions, including prelimbic (PrL), cingulate cortex area 1 (Cg1), cingulate cortex area 2 (Cg2), secondary motor cortex (M2), and PAG, relative to bregma coordinates. The dashed red line indicates the significance threshold (*p* = 0.05).

To quantitatively validate and refine heatmap-based observations, we conducted Pearson's correlation analyses across all female-exposure mice, providing precise measurements of how each brain region's activation relates to vocalization (Fig. 4). Consistent with this framework, regions associated with vocal output exhibited positive correlations between tdTomato^+^ cell counts and USV event numbers. Association strength was interpreted based on both the Pearson's correlation coefficient (*r*) and the coefficient of determination (*R*^2^). Brain regions with *r* > 0.7 and *R*^2^ > 0.5 were considered to exhibit strong correlations (Mai et al., 2023).

To assess region-specific relationships, we calculated correlations between tdTomato^+^ cell counts and USV event numbers for each brain region. For tdTomato signals (Fig. 4*A*), positive correlations were identified in four brain regions: PrL (*r* = 0.685; *R*^2^ = 0.469; *p* = 0.042), Cg1 (*r* = 0.683; *R*^2^ = 0.466; *p* = 0.042), rCPu (*r* = 0.850; *R*^2^ = 0.712; *p* = 0.004), and cPAG (*r* = 0.834; *R*^2^ = 0.696; *p* = 0.020). Among these, rCPu and cPAG met criteria for strong associations (*r* > |0.7| and *R*^2^ > 0.5). Notably, other mPFC subregions, including MO (*r* = 0.647; *R*^2^ = 0.418; *p* = 0.059), IL (*r* = 0.635; *R*^2^ = 0.404; *p* = 0.066), and Cg2 (*r* = 0.587; *R*^2^ = 0.344; *p* = 0.097), exhibited trends toward significance (*p* values approaching 0.05). Conversely, negative correlations were observed in LS (*r* = −0.649; *R*^2^ = 0.4218; *p* = 0.042) and VMH (*r* = −0.693; *R*^2^ = 0.4797; *p* = 0.0386), suggesting that activity in these regions may be associated with USV suppression.

Considering earlier observations from Figure 2, regions such as M2, POA, and LHb showed significantly elevated tdTomato signal densities but lacked statistically significant correlations with USV number (labeled with stars in Fig. 4*A*). These three regions are therefore more likely involved in general courtship-related behaviors rather than direct USV production.

Since behaviorally relevant neurons often cluster within functionally specialized subregions, we further subdivided significant brain regions to examine spatial specificity (Fig. 4*B*). For example, Pearson's correlation analysis of tdTomato expression across a 1.20 mm PAG extent revealed that the site located ∼4.6–4.8 mm posterior to the bregma showed the strongest correlation with USV number. This suggests that neurons responsible for vocal production may concentrate in this area, consistent with previous findings (Tschida et al., 2019). Similar analysis was applied to PrL, M2, Cg1, and Cg2, which showed significant differences in Figure 2. The strongest correlations were found at ∼1.96 mm anterior to the bregma in PrL, 2.00 mm in M2, 0.94 mm in Cg1, and 0.34 mm in Cg2. Notably, the Cg1 finding aligns with a previous report showing that intracortical microstimulation at similar coordinates induces USV production (Gan-Or and London, 2023).

### Vocalization-induced tdTomato expression in the medial striatum is primarily localized within striosome compartments

Based on the above findings, prefrontal regions (e.g., PrL, Cg1) and rCPu (striatum) emerge as strong candidates involved in USV production. These findings suggest the corticostriatal circuit may serve as a key pathway underlying vocalization. The striatum consists of two neurochemical compartments, patch/striosome and the matrix (Pert et al., 1976; Graybiel, 1990; Gerfen, 1992; Graybiel and Matsushima, 2023). Notably, the mPFC preferentially innervates striosomal compartments in the medial striatum (Gerfen, 1984; Nisenbaum et al., 1998; Friedman et al., 2015; Hunnicutt et al., 2016; Choi et al., 2023). We therefore examined whether USV-associated neuronal activity exhibited a compartment-specific pattern in the striatum with a potential bias toward limbic-associated striosomes.

**Figure 5.**
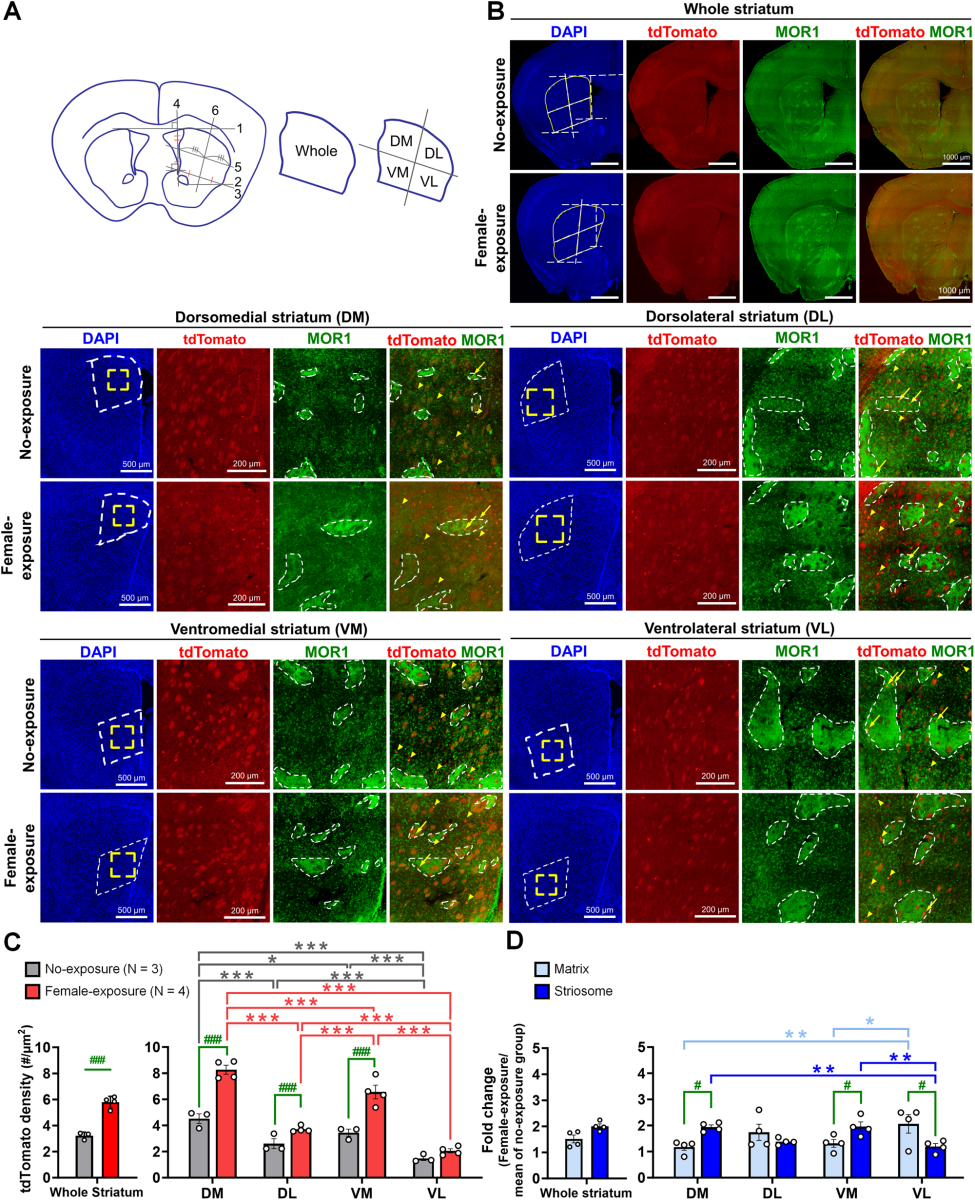
Regional segmentation and compartment-specific activation patterns in the dorsal striatum following female exposure. ***A***, Schematic of the dorsal caudoputamen (CP) segmentation strategy. The CP was divided into four quadrants based on geometric landmarks. First, a reference tangent line (Line 1) connected the dorsal edges of the bilateral CP. Subsequent boundaries were defined relative to this reference: Line 2 originated from the anterior commissure parallel to Line 1; Line 3 connected the lateral CP boundary to the dorsal tip of the nucleus accumbens shell (NAcs); and Line 4 extended vertically from the NAcs perpendicular to Line 1. The internal boundaries (Lines 5 and 6) were then determined by the midpoints of the reference lines. Finally, the CP was delineated into DM, DL, VM, and VL subregions based on these internal borders. ***B***, tdTomato^+^ neurons are identified in distinct striatal compartments by double labeling with MOR1. Nuclei were counterstained with DAPI (blue) to delineate striatal subregions (dashed lines in DAPI images). Yellow boxed areas are shown at higher magnification. tdTomato^+^ neurons (red) within striosomes were identified in MOR1^+^ regions (green; dashed outlines), whereas those in the matrix were located outside MOR1^+^ regions. Arrows and arrowheads indicate the tdTomato^+^ neurons in striosomes and the matrix, respectively. ***C***, Quantification of tdTomato^+^ neuron density across the whole striatum and subregions in the no-exposure and female-exposure groups. Two-way ANOVA revealed significant main effects of exposure and subregions. Female exposure significantly increased tdTomato^+^ cell density in the whole striatum and specifically in the DM, DL, and VM subregions compared with the no-exposure group. Among subregions, more tdTomato-labeled activated neurons were observed in DM and VM. ***D***, Quantification of tdTomato^+^ cell density in each compartment of the female-exposure group, normalized to the mean of the corresponding compartment within the same subregion of the no-exposure group. Two-way ANOVA revealed significant main effects of compartment and subregion. tdTomato^+^ cell density was higher in the striosomal compartment than in the matrix within DM and VM, whereas the matrix compartment showed higher density than the striosome in VL. *^, #^*p* < 0.05; **^, ##^*p* < 0.01; ***^, ###^*p* < 0.001.

We divided the striatum into four subregions: dorsomedial (DM), dorsolateral (DL), ventromedial (VM), and ventrolateral (VL; Morigaki et al., 2020; Fig. 5*A*). We then quantified tdTomato signal densities in these subregions (Fig. 5*B*). DM (4.521 ± 0.369 vs 8.265 ± 0.344; *p* < 0.001), DL (2.604 ± 0.379 vs 3.705 ± 0.117; *p* < 0.05), and VM (3.442 ± 0.268 vs 6.560 ± 0.526; *p* < 0.05) showed significant increases in tdTomato density compared with the non-USV group (Fig. 5*C*; Extended Data Table 2-1). Notably, tdTomato signal level elevation varied, with the DM subregion showing nearly a twofold increase, whereas DL displayed only modest changes.

To further examine compartment-specific activation, we performed immunostaining for mu-opioid receptor 1 (MOR1), a marker of the striosome compartment (Fig. 5*B*,*D*; Extended Data Table 2-1; Pert et al., 1976; Graybiel and Ragsdale, 1978). In medial striatal regions, the number of tdTomato^+^ cells was significantly higher in striosomes than in matrix compartments (DM, 1.938 ± 0.083 striosome vs 1.172 ± 0.125 matrix; *p* < 0.01; VM, 1.954 ± 0.182 striosome vs 1.312 ± 0.150 matrix; *p* < 0.05). In contrast, lateral regions showed an opposite pattern, with higher tdTomato^+^ cell numbers in matrix compartments; however, this difference reached statistical significance only in the VL subregion (1.192 ± 0.114 striosome vs 2.060 ± 0.355 matrix; *p* < 0.05). These findings indicate that vocalization-related neurons are enriched in the medial striatum, particularly within striosomal compartments of the DM CPu.

## Discussion

Our study employed a double-labeling approach to provide an unbiased, whole-brain map of neuronal ensembles activated during social interaction and USV production in male mice. The findings provide convergent evidence that a distributed network of cortical, striatal, and brainstem regions is recruited during this complex behavior. Consistent across a series of IEG-based cell density, heatmap, and correlation analyses, the results indicate that the most robustly and consistently activated regions with a positive association with USV production include subregions of the mPFC, rCP, and cPAG. The identification of this core vocalization network not only validates the established role of the brainstem in vocal production (Jürgens, 2009; Tschida et al., 2019) but also reveals a potential contribution of the corticostriatal circuit, particularly striosomal compartments. Furthermore, our study identified candidate regions involved in general social behavior versus those specifically linked to USV output. These findings establish a comprehensive neuroanatomical framework for future investigations into the precise circuit mechanisms underlying mammalian social communication.

### A distributed network for social vocalization

The results of the activity mapping indicate a distributed neural network recruited during courtship and social vocalization in male mice, extending from the forebrain to the midbrain. The combination of a time-locked tdTomato reporter with acute c-Fos immunostaining allowed for the identification of a stable population of consistently active neurons during USV production while also providing a snapshot of the broader activity state during a single behavioral session. The USV correlation analysis is informative in distinguishing brain regions with a direct functional link to vocal output from those involved in general social or courtship behaviors. By testing the hypothesis that USV-related regions should exhibit a clear relationship between their activity level and the number of USVs produced, the analysis provided a critical filter for the large dataset of activated regions. The results consistently identified the PrL, Cg1, rostral rCPu, and cPAG as regions with strong positive correlations to USV count. In contrast, other regions, such as the POA and LHb, showed significant neuronal activation compared with controls but lacked a statistical correlation with USV number, suggesting a role in broader aspects of social interaction or modulation rather than a direct involvement in vocal production.

Evidence from other species indicates that the limbic system is involved in the vocal network. Echolocating bats produce both ultrasonic communication calls and echolocation signals by using partially segregated neural pathways for these distinct vocalization types. The brainstem organization in bats reveals hierarchical gating mechanisms, with the PAG serving functions analogous to rodent systems, while parallel processing in the paralemniscal area provides an audiovocal interface critical for vocalization control (Montoya et al., 2022; Babl et al., 2025). Furthermore, emotional valence and social context may modulate vocal production decisions by incorporating limbic networks into frontal auditory and vocal control fields. For example, basolateral amygdala neurons are selectively responsive to social vocalizations and decode emotional valence, differentiating between threat and appeasement calls. Amygdala microstimulation triggers multimodal outputs, including echolocation, social calls, tachycardia, and hyperventilation, with connections to the frontal auditory field via pathways that bypass traditional midbrain centers (Kobler et al., 1987; Naumann and Kanwal, 2011; Ma and Kanwal, 2014; Babl et al., 2025; Hoerpel et al., 2025). These convergent circuit architectures, ranging from brainstem motor pattern generators through limbic–cortical integration, provide a multiscale framework for interpreting social-vocal decision-making.

Evidence from rodent studies indicates that limbic circuits play a central role in regulating vocal networks. Rodent vocal production is controlled by the hierarchical integration of social and emotional signals that converge on the midbrain PAG (Jürgens, 2002; Arriaga et al., 2012; Tschida et al., 2019). The medial POA functions as a key driver of courtship vocalizations by disinhibiting PAG–USV neurons through local GABAergic circuitry. In contrast, the central–medial boundary of amygdala (AmgC/M) suppresses vocalization under aversive conditions by directly inhibiting PAG–USV neurons (Michael et al., 2020). These interactions form a nested limbic circuit that enables dynamic evaluation of prosocial versus threat-related drives (Xiao et al., 2023).

Furthermore, higher-order cortical regions provide modulatory input to this limbic–vocalization system. The anterior cingulate cortex contributes to vocal modulation (Gan-Or and London, 2023), while the posterior insula acts as a sensorimotor interface linking auditory processing with vocal motor control (Pomberger et al., 2025). Convergent limbic and cortical inputs ultimately gate PAG activity, which activates downstream pattern generators to coordinate phonation (Veerakumar et al., 2023; Park et al., 2024).

**Figure 6.**
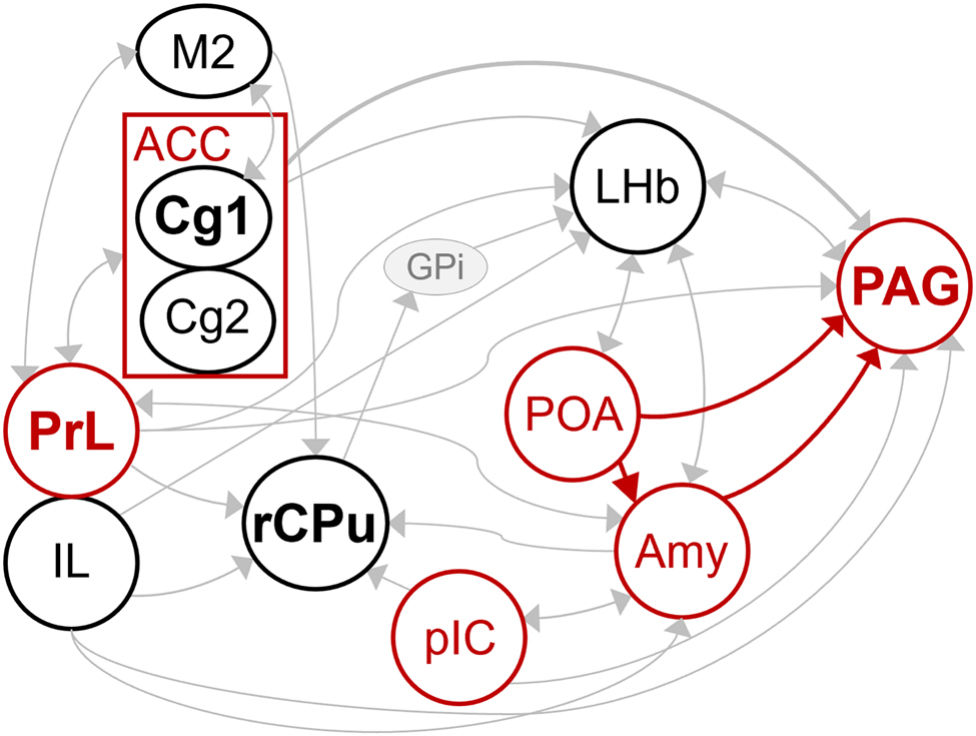
Conceptual forebrain–brainstem network underlying mouse USV. Schematic illustrating the conceptual framework integrating the present findings with established vocal pathways rather than proposing a definitive circuit or causal model. Brain regions highlighted in red indicate nuclei with established roles in vocalization, as supported by prior literature. Red solid arrows represent experimentally validated vocalization pathways. Brain regions shown in black indicate nuclei in which this study identified a significant increase in activated neurons during USV emission. Region names shown in bold denote nuclei in which tdTomato^+^ cell density exhibited a significant positive correlation with the number of USVs produced. Thin gray lines indicate anatomically confirmed projections between regions for which a functional relationship to USV production has not yet been directly tested. Notably, the GPi (shaded in gray) has not been directly examined in the context of vocalization but serves as a key relay through which striatal outputs are conveyed to the LHb. M2, secondary motor cortex; ACC, anterior cingulate cortex; Cg1, cingulate cortex, area 1; Cg2, cingulate cortex, area 2; PrL, prelimbic cortex; IL, infralimbic cortex; rCPu, caudate–putamen, rostral; pIC, posterior insular cortex; POA, preoptic area; Amy, amygdala; PAG, periaqueductal gray.

Within this hierarchical organization, our mapping data suggest that cortical regions such as the PrL, IL, Cg1, and M2 may serve as forebrain coordinators for contextual evaluation and action selection, whereas striosomes and the LHb may interface with this system via basal ganglia pathways that have been implicated in value-based trade-off and motivational processing (Friedman et al., 2015; Bloem et al., 2017, 2022; Fig. 6). These observations point to the involvement of additional limbic- and forebrain-related nodes in the vocalization network, which are discussed individually below.

### Vocal behavior emerges from interacting circuits with distinct roles

A key strength of the USV correlation analysis is its ability to functionally dissociate brain regions involved in general social interaction from those specifically related to USV production. Regions such as the POA and LHb showed significantly elevated tdTomato or c-Fos cell density compared with controls, indicating a role in courtship behavior; however, they lacked a significant correlation with the number of USVs produced. This suggests these regions, while active during the social task, may be part of a broader network for sexual and social arousal that does not directly drive vocal output. This distinction is critical for building a precise neuroanatomical map of the vocalization circuit.

Conversely, the analysis revealed a distinct set of regions that showed negative correlations with USV count, including the LS and VMH. This finding suggests that these areas may be part of a vocal suppression or gating circuit of social behaviors (Lin et al., 2011). It has been shown that inhibiting neuronal activity in the VMH can suppress aggression, and VMH neurons that are activated during attack are inhibited during mating (Lin et al., 2011). The negative correlation observed here is consistent with a model in which a decrease in the suppressive influence of the VMH allows for the robust expression of social-communicative behavior, such as USV production.

### The PAG as a conserved vocal motor hub

The present study shows that cPAG exhibits a strong and consistent pattern of elevated neuronal activity across all three experimental datasets—tdTomato-only, c-Fos-only, and double-labeled cell density analyses. The most compelling evidence for its role in USV production comes from the correlation analysis, where the cPAG exhibited a strong positive correlation between neuronal activity and the number of USVs produced, meeting the study's criteria for a “USV production-related” region (*r* = 0.834; *R*^2^ = 0.696; *p* = 0.020).

This result is consistent with a large body of literature demonstrating that the PAG acts as a final common pathway for vocal output across diverse vertebrate species (Kittelberger et al., 2006; Jürgens, 2009). Previous studies have shown that neurons in the cPAG are required for USV production in mice (Michael et al., 2020). The spatial analysis of tdTomato expression showed that the strongest correlations with USV count were localized to a specific subregion ∼4.6–4.8 mm posterior to the bregma, a finding that aligns with previous anatomical and functional mapping efforts (Tschida et al., 2019). The spatial specificity within the cPAG supports the influential model that functionally distinct vocalizations are gated by anatomically distinct subsets of PAG neurons (Tschida et al., 2019). The identification of this well-known vocal hub with our methodology validates the overall experimental approach and provides a strong foundation for interpreting the more novel findings related to the forebrain circuitry.

### The mPFC is engaged in neural circuits of social vocalization

The whole-brain mapping data suggest a distributed forebrain network that works in concert with the PAG to govern USV production. Subregions of the mPFC, including the PrL, IL, Cg1, and M2, were robustly activated by social interaction, as indicated by significant increases in tdTomato and c-Fos cell density compared with controls. Importantly, the PrL and Cg1 showed a positive correlation between neuronal activity and USV count (PrL, *r* = 0.685; *p* = 0.042; Cg1, *r* = 0.683; *p* = 0.042). This pattern suggests that these areas are not simply activated by general social interaction but are directly involved in the production or modulation of vocal output.

This finding aligns with the mPFC's established role as an integrative center for motivated social and emotional behaviors (Wang et al., 2011; Grossmann, 2013). The mPFC receives inputs from a wide range of cortical and subcortical areas and projects to multiple output structures, including nuclei that we identified in this study as involved in vocalization, such as the striatum, LHb, and PAG. It is possible that the mPFC serves as a critical node in the vocalization circuit, translating an animal's internal motivational state and external social context into the appropriate command signals to drive USV production. The finding that Cg1 activation positively correlated with USV count is consistent with a previous report where intracortical microstimulation and optogenetic activation in the anterior cingulate cortex can induce USV production (Gan-Or and London, 2023). This provides a crucial causal link, supporting the functional relevance of the correlational findings. The involvement of the PrL in both vocal production (as shown here) and in the reception of USVs in other contexts suggests a potential vocal-auditory integration loop that could facilitate social communication (Asaba et al., 2017; Pomberger et al., 2025). Note that a previous study demonstrated that microstimulation of posterior PrL or Cg2 is sufficient to induce USV in rats with longer latency, implicating the mPFC in vocal initiation (Bennett et al., 2019). Additionally, future work should clarify whether the PrL contributes to the direct volitional pathway to the nucleus ambiguus or instead acts primarily within an innate limbic-driven pathway for vocalization (Jürgens, 2009; Arriaga et al., 2012; Nieder and Mooney, 2020). It also remains to be determined whether the PrL, similar to the insular cortex (Pomberger et al., 2025), contains distinct neuronal subpopulations responsible for the reception and expression of vocalization and for coordinating inputs from other sensory modalities.

### A potential involvement of striosomal compartments in social vocalization

One of the most interesting findings of this study is the robust activation of the rCPu and its striking correlation with USV production (*r* = 0.850; *R*^2^ = 0.712; *p* = 0.004). This strong association suggests the critical role of the corticostriatal circuit in mouse vocalization, a pathway that has previously been primarily linked to learned, rather than innate, vocal behaviors (Scharff and Nottebohm, 1991; Jarvis, 2004; Aronov et al., 2008). Consistently, previous studies have also reported increased numbers of c-Fos–expressing cells in the anterodorsal striatum following USV emission in mice, supporting the engagement of striatal circuits during vocalization (Arriaga et al., 2012). Furthermore, a detailed neuroanatomical analysis revealed that vocalization-related neuronal activity within the medial striatum was preferentially localized within the striosome compartments rather than the surrounding matrix. The striatum is anatomically and functionally divided into the two neurochemical compartments: striosomes receive preferential input from limbic cortical regions, such as the mPFC, and are presumably involved in emotional and motivational processing, while the matrix is more associated with sensorimotor and associative functions (Crittenden and Graybiel, 2011; Friedman et al., 2015). The finding that USV-related activity is enriched in striosomes of the medial striatum suggests that the vocalization signal is routed through the striatum's limbic–affective pathway, consistent with the highly motivated nature of courtship behavior. This provides a clear functional segregation within the striatum related to USV production, suggesting that the USV-elicited neuronal activity is driven by motivation and reward, not just motor execution.

Notably, the DM striatum receives dense projections from the PrL, and PrL preferentially innervates the striosome compartment (Gerfen, 1984; Nisenbaum et al., 1998; Friedman et al., 2015; McGregor et al., 2019; Choi et al., 2023). It raises the possibility that activation of the PrL–striosome pathway may be engaged in vocal socialization. This PrL–striosome circuit may represent a limbic–motor circuit for integrating emotion and motivation for modulating vocal motor behaviors.

The finding of a USV-related corticostriatal circuit, specifically involving the striosome compartments of medial striatum, provides a potential evolutionary link between the innate mouse USV system and the more complex, learned vocal systems in humans and songbirds (Scharff and Nottebohm, 1991; Jarvis, 2004; Aronov et al., 2008). In these species, the corticostriatal pathway is known to be critical for vocal learning and motor sequencing (Scharff and Nottebohm, 1991; Jarvis, 2004; Aronov et al., 2008). While mouse USVs are largely considered innate and not dependent on auditory feedback for development (Holy and Guo, 2005; Arriaga et al., 2012), the involvement of a homologous circuit raises a compelling hypothesis. This circuit in mice may not be involved in learning new vocalizations but rather in modulating innate ones based on reinforcement contingencies and social context, a more primitive but related function (Asaba et al., 2017).

### Potential role of LHb in integrating forebrain and basal ganglia signals during vocal behavior

Given the involvement of forebrain circuits in shaping vocal behavior, the LHb may represent a potential node at the interface between evaluative processing and the control of vocal actions. The mPFC sends prominent projections to the LHb, forming a top–down pathway thought to be essential for strategy switching (Mizumori and Baker, 2017). The mPFC signals the LHb to evaluate whether the current behavioral decision aligns with the animal's internal state and whether it should be maintained or adjusted. Concurrently, the LHb serves as a convergence point for basal ganglia outputs, receiving signals from the striatum via the globus pallidus internus (GPi)/entopeduncular nucleus (EP; Shabel et al., 2012; Hong et al., 2019). Importantly, this striatal input to the LHb is thought to arise predominantly from striosomal compartments (Hong et al., 2019). This basal ganglia–habenular pathway is classically associated with encoding negative valence and evaluating action outcomes. By integrating these inputs, the LHb regulates downstream dopaminergic neurons in the ventral tegmental area, either directly or indirectly via the rostromedial tegmental nucleus (RMTg), to signal negative reward prediction errors (Mondoloni et al., 2022).

Our results indicated that LHb activity was significantly elevated during USV emission. However, the increase in the number of activated cells did not correlate with the number of USVs, suggesting that the LHb is unlikely to directly drive vocal output. Instead, the LHb may be responsible for processing a social cost–benefit evaluation, in which the male mouse assesses the safety and appropriateness of vocalizing based on fluctuating internal and external environmental cues. This interpretation is consistent with previous findings that the LHb plays a pivotal role in modulating social preferences and interactions (Benekareddy et al., 2018; Dodis et al., 2026).

Furthermore, the increased activation observed in the DMS during USV emission suggests that the LHb may be engaged in vocal control via striosome-mediated modulation (Hong et al., 2019). Ultimately, such complicated circuit dynamics allow the LHb to regulate action outcomes via the dopaminergic system, ensuring that social-vocal behaviors are adaptively tuned to the current context.

### Limitations of the study

Our study has limitations. To identify brain regions that are activated and contribute to vocalization in adult male mice, we induced USV production in male mice by social encounters with females. Although our primary focus is on the vocalization component of behavior, it is important to note that multiple types of information are simultaneously processed in the brain during social interactions. These include, for example, sex discrimination and social exploratory behavior, such as anogenital sniffing and close following before mating behavior. Notably, we did not quantitatively assess behavioral or neural responses of the female mice during these interactions. Therefore, we cannot exclude the possibility that some of the observed neural activations in males may be influenced by the females' responses to male USVs, rather than reflecting vocal production per se*.* Additionally, neuronal activities related to stress or arousal may also be engaged. These related stimuli can also evoke widespread activation across the brain, independent of vocalization itself. Therefore, the activity-based whole-brain mapping reveals candidate regions associated with USV production but does not provide direct evidence of causal involvement in vocalization. Despite these limitations, our study provides groundwork for future research aimed at dissecting the dynamic interplay between cortical, striatal, and brainstem circuits that underpins mammalian social communication.

## Synthesis

Reviewing Editor: Sam Golden, The University of Washington Decisions are customarily a result of the Reviewing Editor and the peer reviewers coming together and discussing their recommendations until a consensus is reached. When revisions are invited, a fact-based synthesis statement explaining their decision and outlining what is needed to prepare a revision will be listed below. The following reviewer(s) agreed to reveal their identity: Cynthia Kelm-Nelson.

Dear Authors,

Your manuscript has been reviewed by two experts in the field, who are both enthusiastic about the manuscript but identified several areas where revisions would be appropriate and strengthen the final version. Please see their comments below. I look forward to viewing the revised manuscript.

Reviewer 1:

This is a well-written and compelling manuscript that quantitatively examines brain-region activity during courtship vocalizations in mice. The discussion of potential evolutionary links and the modulation of innate vocal behaviors by social context is particularly engaging and relevant to ongoing debates about innate versus learned vocal communication. I am enthusiastic about this work; my comments are relatively minor but should help strengthen the manuscript.

1. The description of striatal organization would be better suited to the Introduction rather than the Results section. Moving this material earlier would help situate readers before the data are presented.

2. The sample size is not clearly stated in the Methods, and the use (or absence) of control groups is also not mentioned. Please consider adding this. Directional hypotheses would also be helpful.

3. Many methodological details currently appear in the Results section. This makes it difficult to follow the experimental sequence and interpret the findings. I strongly recommend reorganizing the manuscript to ensure all methods are described in the appropriate Methods section.

4.Please double-check that the IHC description and figure labeling correspond accurately. Additionally, it would be helpful to clarify the rationale for using MOR1.

5.How many raters manually delineated the 25 brain regions, and across how many subjects? Information about inter-rater consistency or training would also strengthen confidence in the quantification.

6. Were mice engaged in any other behaviors during testing (e.g., feeding, drinking)? Also, were all recorded vocalizations affiliative, or were other categories (distress) observed?

Reviewer 2:

In this study, the authors used a double-labelling strategy that combined genetic activity tagging (TRAP2) and c-Fos immunohistochemistry to generate a whole-brain map of neuronal ensembles activated during courtship-induced USV production in adult male mice. The study reports new findings on USV-related neurons in the striatum that are preferentially localized to striosomal compartments within the multi-level distributed nature of the neural architecture underlying mammalian social communication. Its significance relies on using the relatively novel double-labeling strategy to reveal a potential limbic-motor interface for integrating social motivation with vocal motor output. These data have the potential to open new areas of investigation related to social vocal behavior.

Statements that corticostriatal limbic-motor circuits modulate or integrate innate vocalization contingent on emotional and social contexts, however, are vague and these along with a limited scope of the Discussion indicate a major weakness of the study as presented. Another limitation as stated by the authors relates to the separation of social motivational vs. vocalization roles of the newly identified brain regions. Nevertheless, being more of an exploratory study, it does provide new, albeit correlational, findings that will need to be tested experimentally to gain a better understanding of their true significance.

Below I list some problematic statements/text that should be modified to improve presentation of the data.

Last sentence of Introduction: " ... our study provides new insights into the evolutionarily conserved social vocalization." - did you mean to state: "social vocalization network?

In methods, why were 30 μm free-floating coronal sections spaced approximately 360 μm apart? This could lead to missing areas of neuronal activity in a study where the main focus was mapping brain activity. Please explain.

The Results section: "Unbiased whole-brain mapping of courtship USV-related neurons in mice" should be moved to the Methods.

The Discussion appears limited in its scholarship. Its scope and comprehensiveness can be improved by incorporating pioneering and substantial scientific literature on both social vocal and auditory circuits studied within the brainstem of echolocating bat species (that also produce ultrasonic vocalizations) and more importantly within the limbic (amygdala) parts of the brain, including the forebrain (frontal auditory field and anterior cingulate). This additional effort will add depth to the interpretation of data and enhance significance of this study by allowing authors to possibly make more definitive statements related to their findings. For example, referring to striatum and limbic/prelimbic brain regions generally does not inform the reader about a clear network level interpretation of the data and the role the newly discovered regions of interest may play in modulating social behavior and/or vocal output.

In Figure 3, the Lhb shows significance for tdTomato+ (B1) or c-Fos+ (B2) cell densities labeling. This is interesting given the importance of the habenula in attention and suppression of dopaminergic activity as part of the anti-reward system. Please comment on these findings under Discussion, preferably within a separate section. Could this brain region be activated based on the behavior of the female in response to the vocalizations? Was the behavior of the female tracked in any way?

Table 1 should be moved to the supplementary section. In Table 1, instead of "Data Structure", Say "Normality present" and list "Yes" or "No". This will provide space to add another column with the name of the brain region in addition to its abbreviation. This will be more informative and serve as a quick reference so one does not have to search for the meaning of each abbreviation in the text.

Figure 2 also should be moved to the supplementary section. One or two panels of important new results related to the striatum and/or forebrain could be retained here as examples. Looking at pages of serial transverse sections of the brain is not very informative to readers outside the field and sometimes neurophysiologists/ethologists within the field. Instead, please create a final summary schematic with the brain regions identified to be part of the vocal motor circuit preferably within a sagittal section of the mouse brain. Label known vs. putative connections. This will also help to identify what is the new information gained from this study.

In Figure 4, what do the stars for POA and LHb panels indicate?

In legend for figure 5, statements like "Draw a reference tangent line (line 1) connecting the dorsal edges of the right and left CP." need to be appropriately modified.
