# Supplementary material for: Whole-Brain Mapping of Neuronal Activity Associated with Vocal Socialization Behaviors in Adult Mice
Source: eNeuro. 2026 May 14;13(5):ENEURO.0400-25.2026. doi: 10.1523/ENEURO.0400-25.2026 (PMC13183370; doi:10.1523/ENEURO.0400-25.2026)
Supplement: Table 2-1 — Statistical Table. Download Table 2-1, DOCX file. [file eneuro-13-ENEURO.0400-25.2026-s002.docx]

**Table 2-1. Statistical Table**

|  | **Normality present** | **Type of test** | **95% confidence interval** | |
| --- | --- | --- | --- | --- |
|  |  |  | **Lower Bound** | **Upper Bound** |
| Fig. 2B1 | |  |  |  |
| Lateral orbital cortex (LO) | Yes | Student's *t-*test | 17.984 | 30.159 |
| Ventral orbital cortex (VO) | Yes | Student's *t-*test | 23.641 | 37.549 |
| Medial orbital cortex (MO) | Yes | Student's *t-*test | 15.879 | 24.411 |
| Prelimbic cortex (PrL) | Yes | Student's *t-*test | 14.168 | 22.602 |
| Infralimbic cortex (IL) | Yes | Student's *t-*test | 16.086 | 24.931 |
| Dorsal peduncular cortex (DP) | Yes | Student's *t-*test | 11.709 | 18.644 |
| Cingulate cortex, area 1 (Cg1) | Yes | Student's *t-*test | 14.056 | 22.036 |
| Cingulate cortex, area 2 (Cg2) | Yes | Student's *t-*test | 17.225 | 25.491 |
| Primary motor cortex (M1) | Yes | Student's *t-*test | 6.357 | 10.766 |
| Secondary motor cortex (M2) | Yes | Student's *t-*test | 10.126 | 16.583 |
| Caudate-putamen, rostral (rCPu) | Yes | Student's *t-*test | 2.809 | 4.776 |
| Caudate-putamen, caudal (cCPu) | Yes | Student's *t-*test | 1.938 | 3.379 |
| Caudate-putamen, tail (tCPu) | Yes | Student's *t-*test | 3.611 | 6.727 |
| Lateral septum (LS) | Yes | Student's *t-*test | 12.061 | 19.364 |
| Piriform cortex (Pir) | No | Mann-Whitney *U* test | 18.154 | 25.006 |
| Insular cortex (InC) | Yes | Student's *t-*test | 8.506 | 13.260 |
| Centromedial amygdala (CeM) | Yes | Student's *t-*test | 15.093 | 21.469 |
| Basolateral amygdala (BLA) | Yes | Student's *t-*test | 12.859 | 18.572 |
| Centromedial thalamus (CMT) | Yes | Student's *t-*test | 19.717 | 28.069 |
| Periventricular thalamus (PVT) | Yes | Student's *t-*test | 62.043 | 77.127 |
| Ventromedial hypothalamus (VMH) | Yes | Student's *t-*test | 8.392 | 15.673 |
| Preoptic area (POA) | Yes | Student's *t-*test | 13.323 | 17.994 |
| Lateral habenula (LHb) | Yes | Student's *t-*test | 9.740 | 14.419 |
| Periaqueductal gray, rostral (rPAG ) | Yes | Student's *t-*test | 13.937 | 18.350 |
| Periaqueductal gray, caudal (cPAG) | Yes | Student's *t-*test | 14.186 | 20.292 |
| Fig. 2B2 | |  |  |  |
| Lateral orbital cortex (LO) | Yes | Student's *t-*test | 18.886 | 29.897 |
| Ventral orbital cortex (VO) | Yes | Student's *t-*test | 25.322 | 39.767 |
| Medial orbital cortex (MO) | No | Mann-Whitney *U* test | 17.396 | 25.527 |
| Prelimbic cortex (PrL) | Yes | Student's *t-*test | 15.619 | 21.321 |
| Infralimbic cortex (IL) | Yes | Student's *t-*test | 17.083 | 26.981 |
| Dorsal peduncular cortex (DP) | No | Mann-Whitney *U* test | 14.116 | 22.838 |
| Cingulate cortex, area 1 (Cg1) | Yes | Student's *t-*test | 13.208 | 18.606 |
| Cingulate cortex, area 2 (Cg2) | Yes | Student's *t-*test | 15.025 | 22.745 |
| Primary motor cortex (M1) | Yes | Student's *t-*test | 3.662 | 6.194 |
| Secondary motor cortex (M2) | Yes | Student's *t-*test | 8.253 | 13.364 |
| Caudate-putamen, rostral (rCPu) | Yes | Student's *t-*test | 2.677 | 4.428 |
| Caudate-putamen, caudal (cCPu) | Yes | Student's *t-*test | 1.837 | 3.007 |
| Caudate-putamen, tail (tCPu) | Yes | Student's *t-*test | 4.301 | 7.571 |
| Lateral septum (LS) | Yes | Student's *t-*test | 11.254 | 15.594 |
| Piriform cortex (Pir) | Yes | Student's *t-*test | 23.514 | 29.257 |
| Insular cortex (InC) | Yes | Student's *t-*test | 8.253 | 14.049 |
| Centromedial amygdala (CeM) | Yes | Student's *t-*test | 5.896 | 8.388 |
| Basolateral amygdala (BLA) | Yes | Student's *t-*test | 11.118 | 16.998 |
| Centromedial thalamus (CMT) | Yes | Student's *t-*test | 12.258 | 17.360 |
| Periventricular thalamus (PVT) | Yes | Student's *t-*test | 36.977 | 49.256 |
| Ventromedial hypothalamus (VMH) | Yes | Student's *t-*test | 10.742 | 21.221 |
| Preoptic area (POA) | Yes | Student's *t-*test | 10.867 | 16.488 |
| Lateral habenula (LHb) | Yes | Student's *t-*test | 8.326 | 13.613 |
| Periaqueductal gray, rostral (rPAG ) | Yes | Student's *t-*test | 14.357 | 18.391 |
| Periaqueductal gray, caudal (cPAG) | Yes | Student's *t-*test | 14.759 | 21.412 |
| Fig. 2C1 | | |  |  |
| Lateral orbital cortex (LO) | No | Mann-Whitney *U* test | 2.892 | 7.255 |
| Ventral orbital cortex (VO) | Yes | Student's *t-*test | 5.113 | 7.994 |
| Medial orbital cortex (MO) | Yes | Student's *t-*test | 2.841 | 4.543 |
| Prelimbic cortex (PrL) | Yes | Student's *t-*test | 2.017 | 4.197 |
| Infralimbic cortex (IL) | Yes | Student's *t-*test | 3.211 | 8.152 |
| Dorsal peduncular cortex (DP) | Yes | Student's *t-*test | 1.830 | 4.368 |
| Cingulate cortex, area 1 (Cg1) | Yes | Student's *t-*test | 1.566 | 5.936 |
| Cingulate cortex, area 2 (Cg2) | Yes | Student's *t-*test | 2.399 | 8.487 |
| Primary motor cortex (M1) | Yes | Student's *t-*test | 0.269 | 1.984 |
| Secondary motor cortex (M2) | Yes | Student's *t-*test | 1.054 | 4.382 |
| Caudate-putamen, rostral (rCPu) | Yes | Student's *t-*test | 0.073 | 0.333 |
| Caudate-putamen, caudal (cCPu) | Yes | Student's *t-*test | 0.180 | 0.456 |
| Caudate-putamen, tail (tCPu) | Yes | Student's *t-*test | 0.327 | 2.091 |
| Lateral septum (LS) | Yes | Student's *t-*test | 3.658 | 7.750 |
| Piriform cortex (Pir) | No | Mann-Whitney *U* test | 6.464 | 11.300 |
| Insular cortex (InC) | Yes | Student's *t-*test | 1.232 | 3.156 |
| Centromedial amygdala (CeM) | Yes | Student's *t-*test | 1.567 | 3.879 |
| Basolateral amygdala (BLA) | No | Mann-Whitney *U* test | 2.344 | 4.490 |
| Centromedial thalamus (CMT) | Yes | Student's *t-*test | 4.262 | 11.200 |
| Periventricular thalamus (PVT) | Yes | Student's *t-*test | 18.109 | 39.364 |
| Ventromedial hypothalamus (VMH) | Yes | Student's *t-*test | 0.635 | 8.385 |
| Preoptic area (POA) | No | Mann-Whitney *U* test | 1.976 | 6.853 |
| Lateral habenula (LHb) | Yes | Student's *t-*test | 1.331 | 3.139 |
| Periaqueductal gray, rostral (rPAG ) | Yes | Student's *t-*test | 4.679 | 8.998 |
| Periaqueductal gray, caudal (cPAG) | Yes | Student's *t-*test | 4.470 | 10.169 |
| Fig. 2C2 | | |  |  |
| Lateral orbital cortex (LO) | Yes | Student's *t-*test | 12.875 | 31.023 |
| Ventral orbital cortex (VO) | No | Mann-Whitney *U* test | 15.889 | 35.127 |
| Medial orbital cortex (MO) | Yes | Student's *t-*test | 13.974 | 25.154 |
| Prelimbic cortex (PrL) | Yes | Student's *t-*test | 11.744 | 23.597 |
| Infralimbic cortex (IL) | Yes | Student's *t-*test | 21.258 | 28.379 |
| Dorsal peduncular cortex (DP) | No | Mann-Whitney *U* test | 12.268 | 21.630 |
| Cingulate cortex, area 1 (Cg1) | Yes | Student's *t-*test | 10.552 | 24.044 |
| Cingulate cortex, area 2 (Cg2) | Yes | Student's *t-*test | 16.691 | 24.245 |
| Primary motor cortex (M1) | Yes | Student's *t-*test | 5.152 | 12.179 |
| Secondary motor cortex (M2) | Yes | Student's *t-*test | 9.044 | 18.954 |
| Caudate-putamen, rostral (rCPu) | Yes | Student's *t-*test | 4.669 | 10.258 |
| Caudate-putamen, caudal (cCPu) | Yes | Student's *t-*test | 5.587 | 10.813 |
| Caudate-putamen, tail (tCPu) | Yes | Student's *t-*test | 4.372 | 16.101 |
| Lateral septum (LS) | Yes | Student's *t-*test | 20.848 | 36.332 |
| Piriform cortex (Pir) | Yes | Student's *t-*test | 15.945 | 28.461 |
| Insular cortex (InC) | Yes | Student's *t-*test | 6.556 | 16.239 |
| Centromedial amygdala (CeM) | No | Mann-Whitney *U* test | 17.126 | 31.890 |
| Basolateral amygdala (BLA) | Yes | Student's *t-*test | 15.255 | 27.367 |
| Centromedial thalamus (CMT) | Yes | Student's *t-*test | 17.517 | 32.271 |
| Periventricular thalamus (PVT) | Yes | Student's *t-*test | 37.450 | 50.711 |
| Ventromedial hypothalamus (VMH) | Yes | Student's *t-*test | 13.007 | 23.579 |
| Preoptic area (POA) | Yes | Student's *t-*test | 12.524 | 30.444 |
| Lateral habenula (LHb) | Yes | Student's *t-*test | 11.757 | 18.791 |
| Periaqueductal gray, rostral (rPAG ) | Yes | Student's *t-*test | 30.565 | 36.771 |
| Periaqueductal gray, caudal (cPAG) | Yes | Student's *t-*test | 27.607 | 40.996 |
| Fig. 5C | | |  |  |
| Whole striatum (CPu) | Yes | Student's *t-*test | 3.368 | 5.998 |
| Dorsomedial (DM) | Yes | Two-way ANOVA | 4.725 | 8.596 |
| Dorsolateral (DL ) | Yes | Two-way ANOVA | 2.567 | 3.899 |
| Ventromedial (VM) | Yes | Two-way ANOVA | 3.518 | 6.929 |
| Ventrolateral (VL) | Yes | Two-way ANOVA | 1.423 | 2.195 |
| Fig. 5D | | |  |  |
| Whole striatum (CPu) | Yes | Student's *t-*test | 1.470 | 2.048 |
| Dorsomedial (DM) | Yes | Two-way ANOVA | 1.176 | 1.935 |
| Dorsolateral (DL) | Yes | Two-way ANOVA | 1.178 | 1.942 |
| Ventromedial (VM) | Yes | Two-way ANOVA | 1.247 | 2.019 |
| Ventrolateral (VL) | Yes | Two-way ANOVA | 1.063 | 2.189 |
