# Supplementary figures and images for: Whole-Brain Mapping of Neuronal Activity Associated with Vocal Socialization Behaviors in Adult Mice
Source: eNeuro. 2026 May 14;13(5):ENEURO.0400-25.2026. doi: 10.1523/ENEURO.0400-25.2026 (PMC13183370; doi:10.1523/ENEURO.0400-25.2026)

**Extended Data Figure 2-1**

**
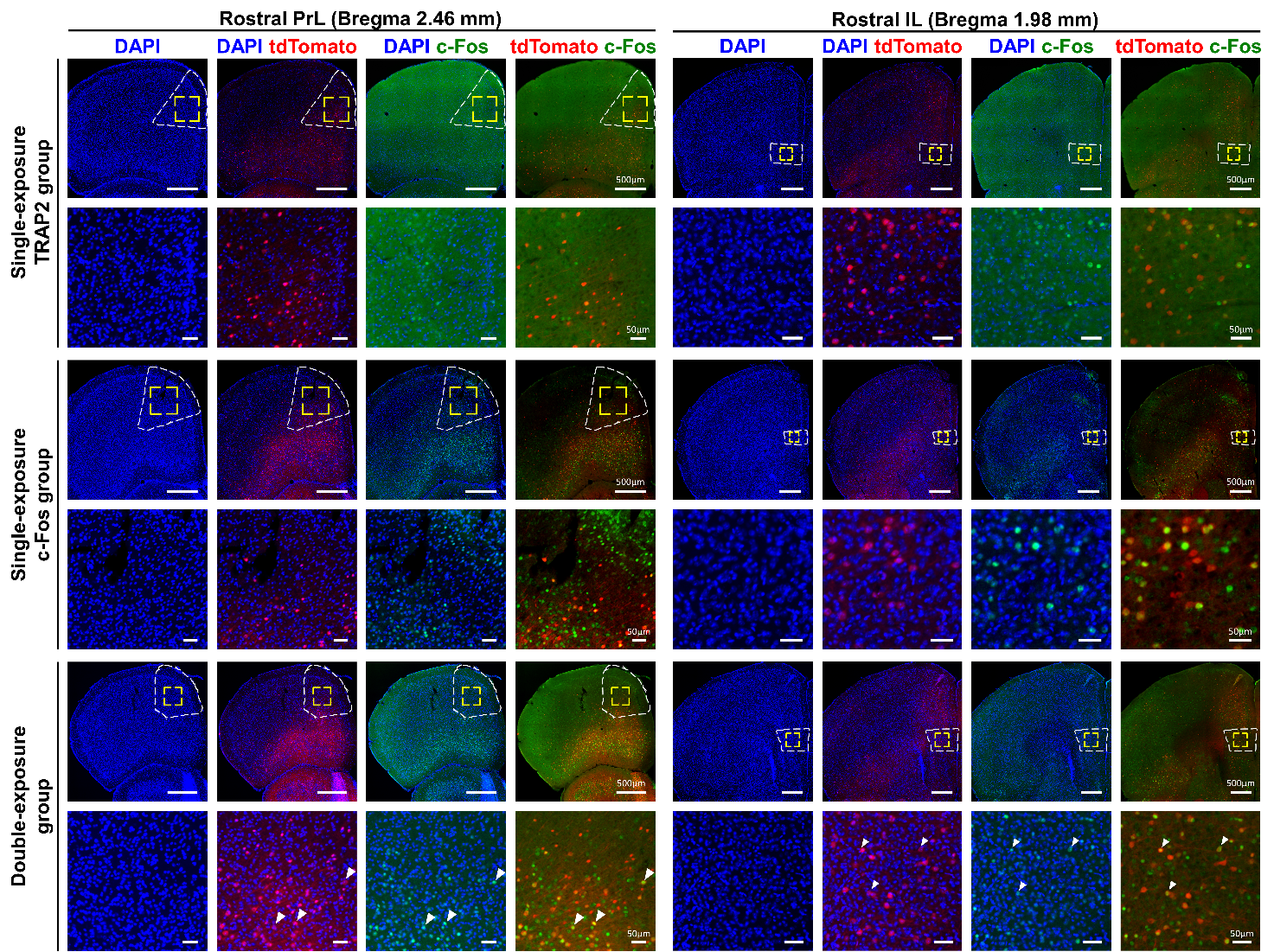
**

**
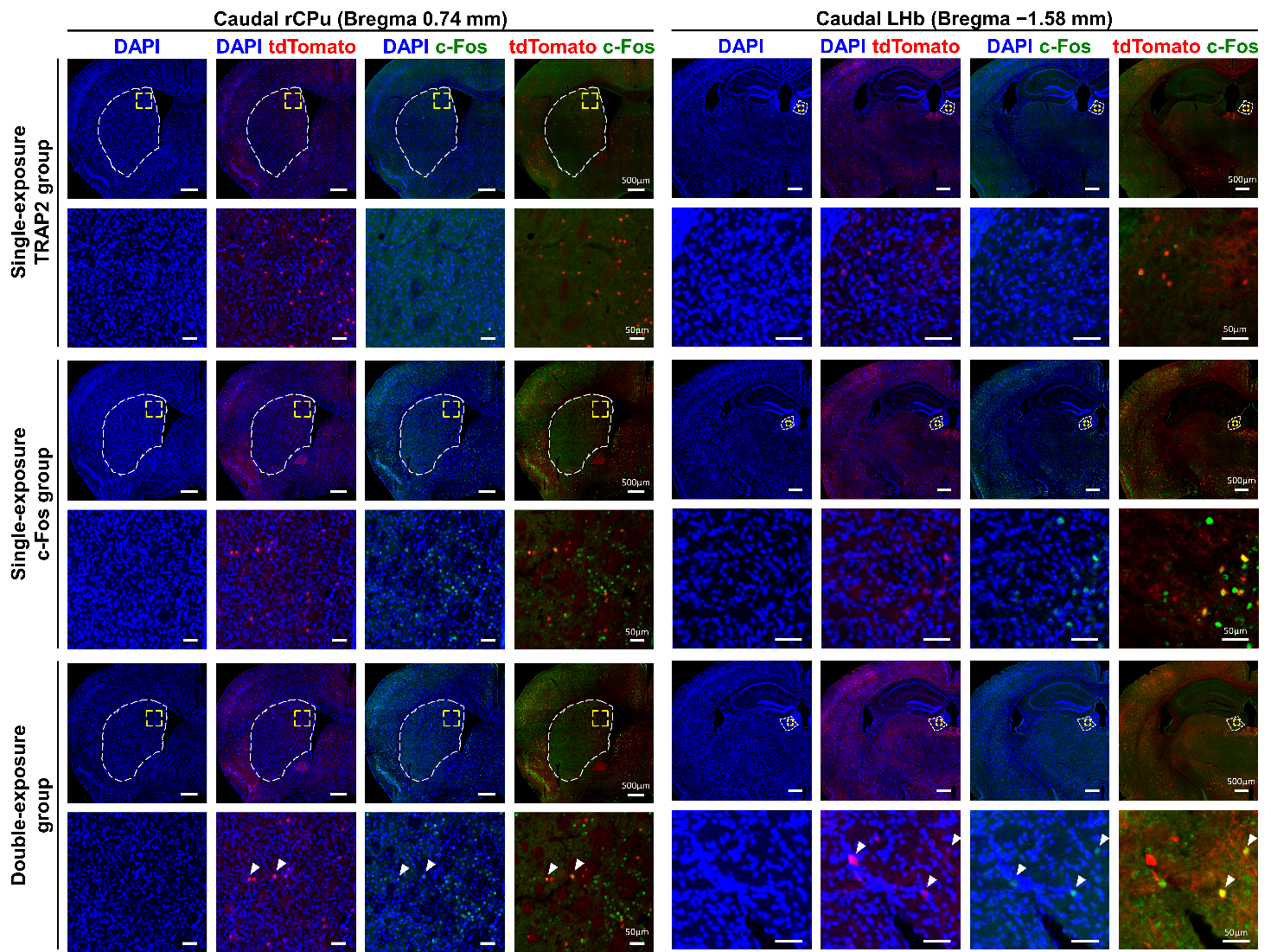
**

**
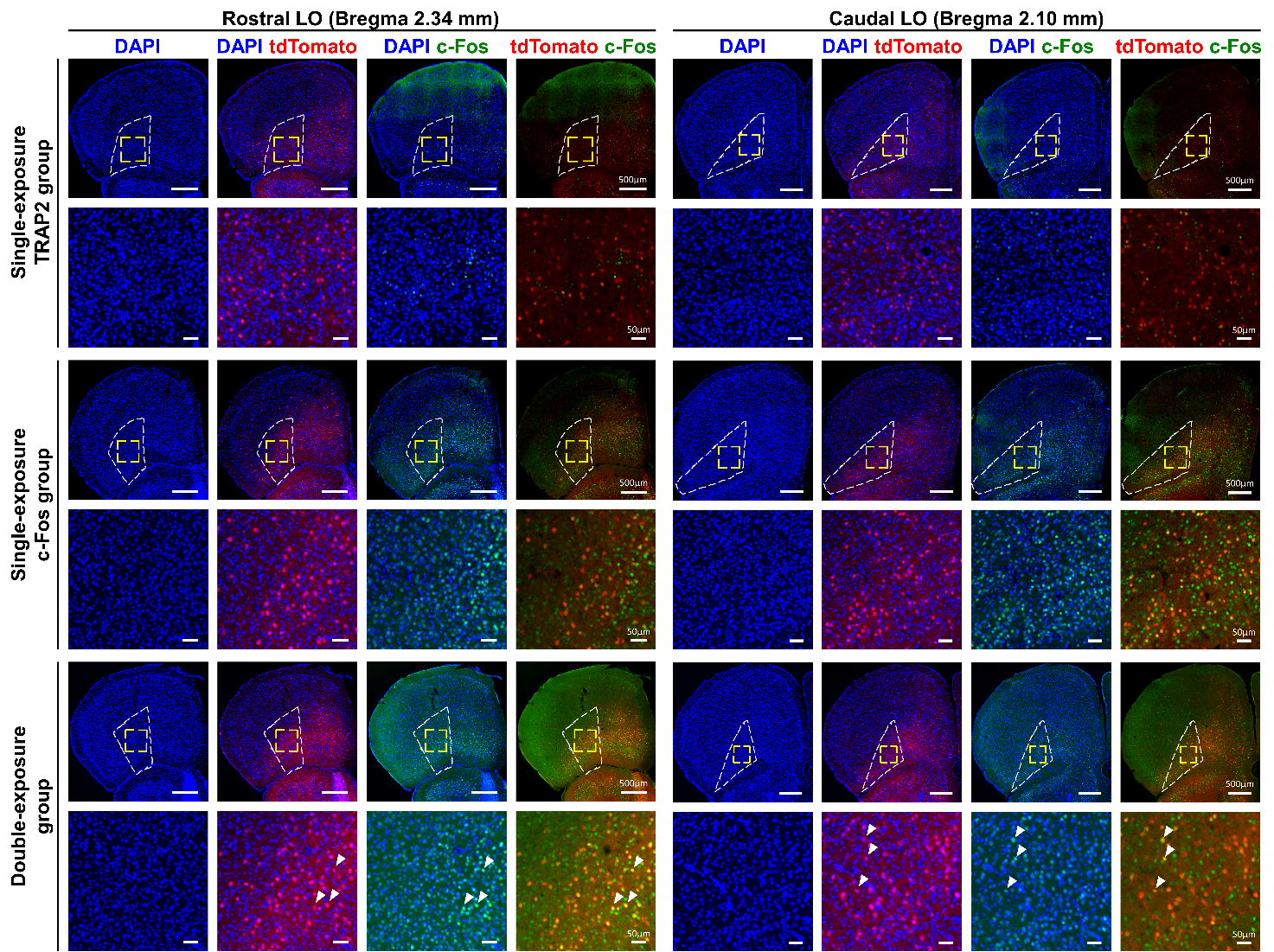
**

**
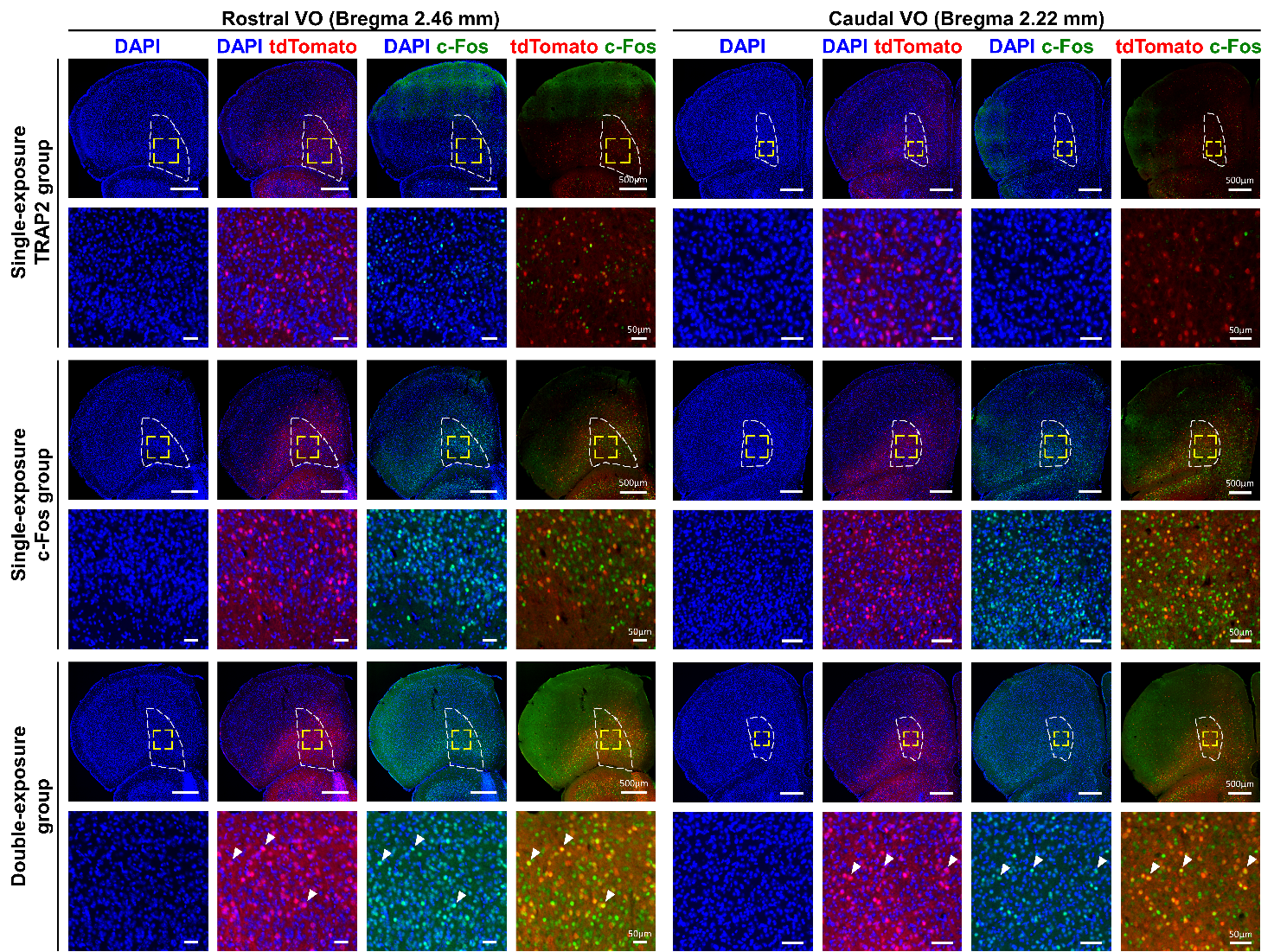
**

**
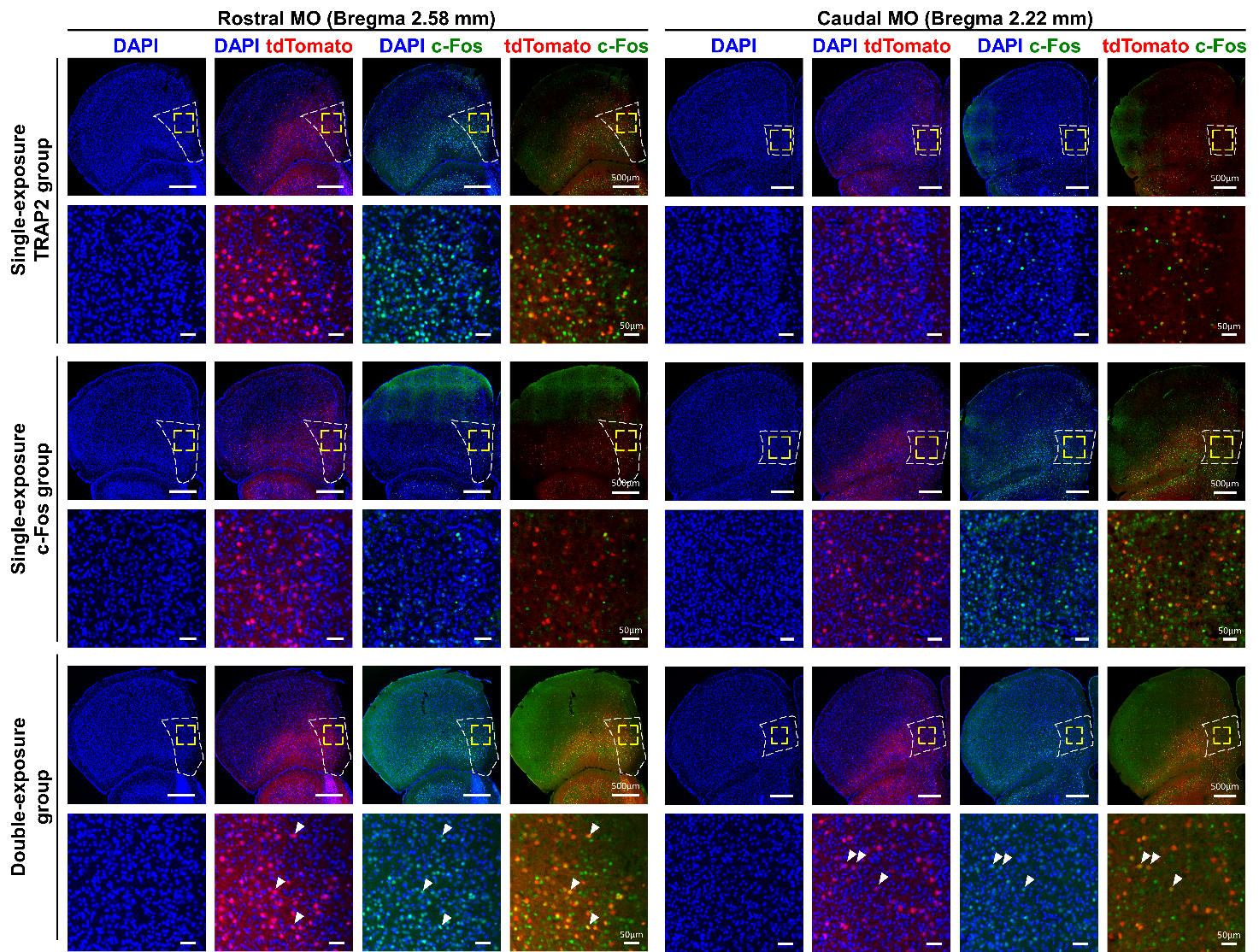
**

**
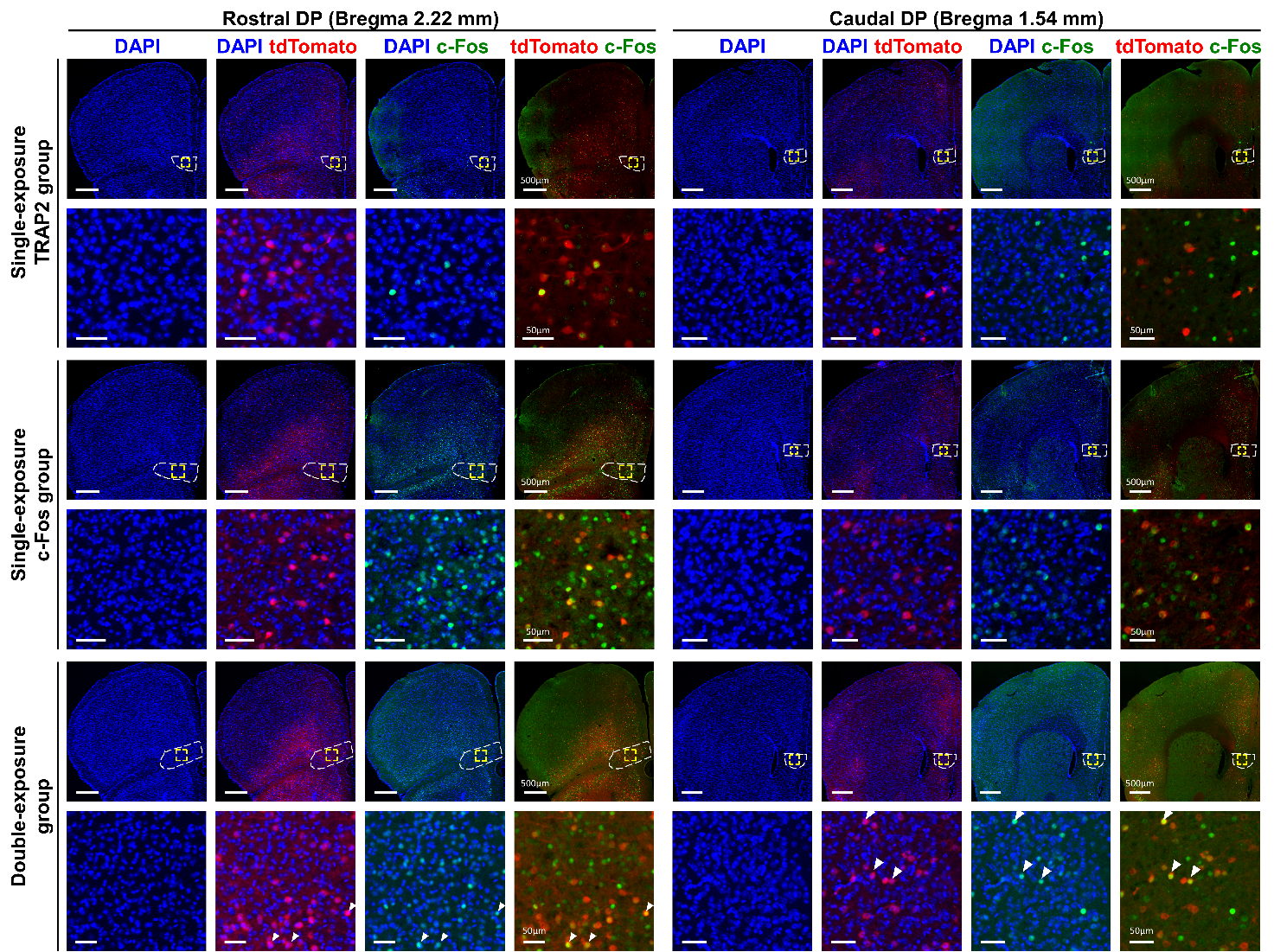
**

**
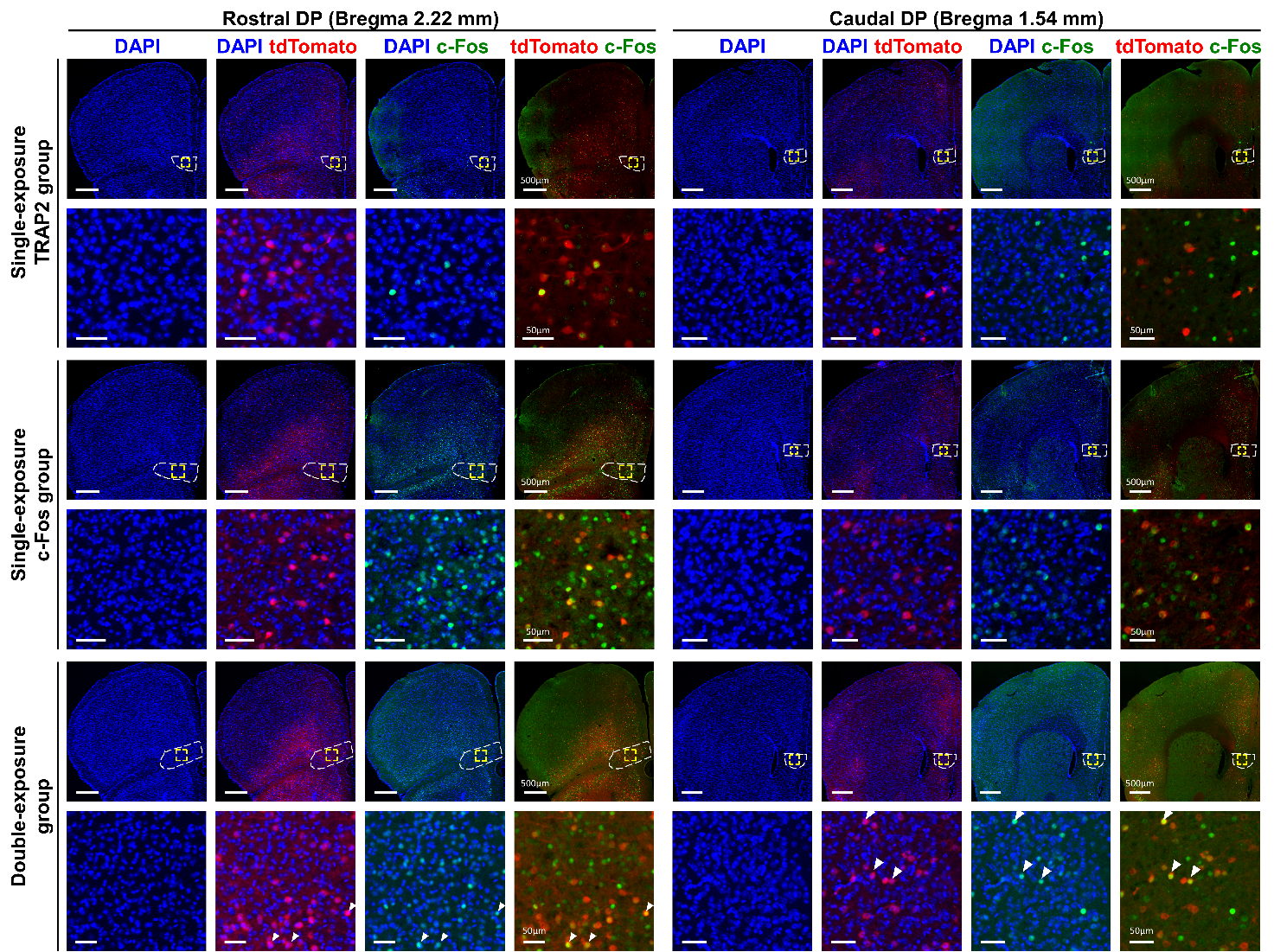
**

**
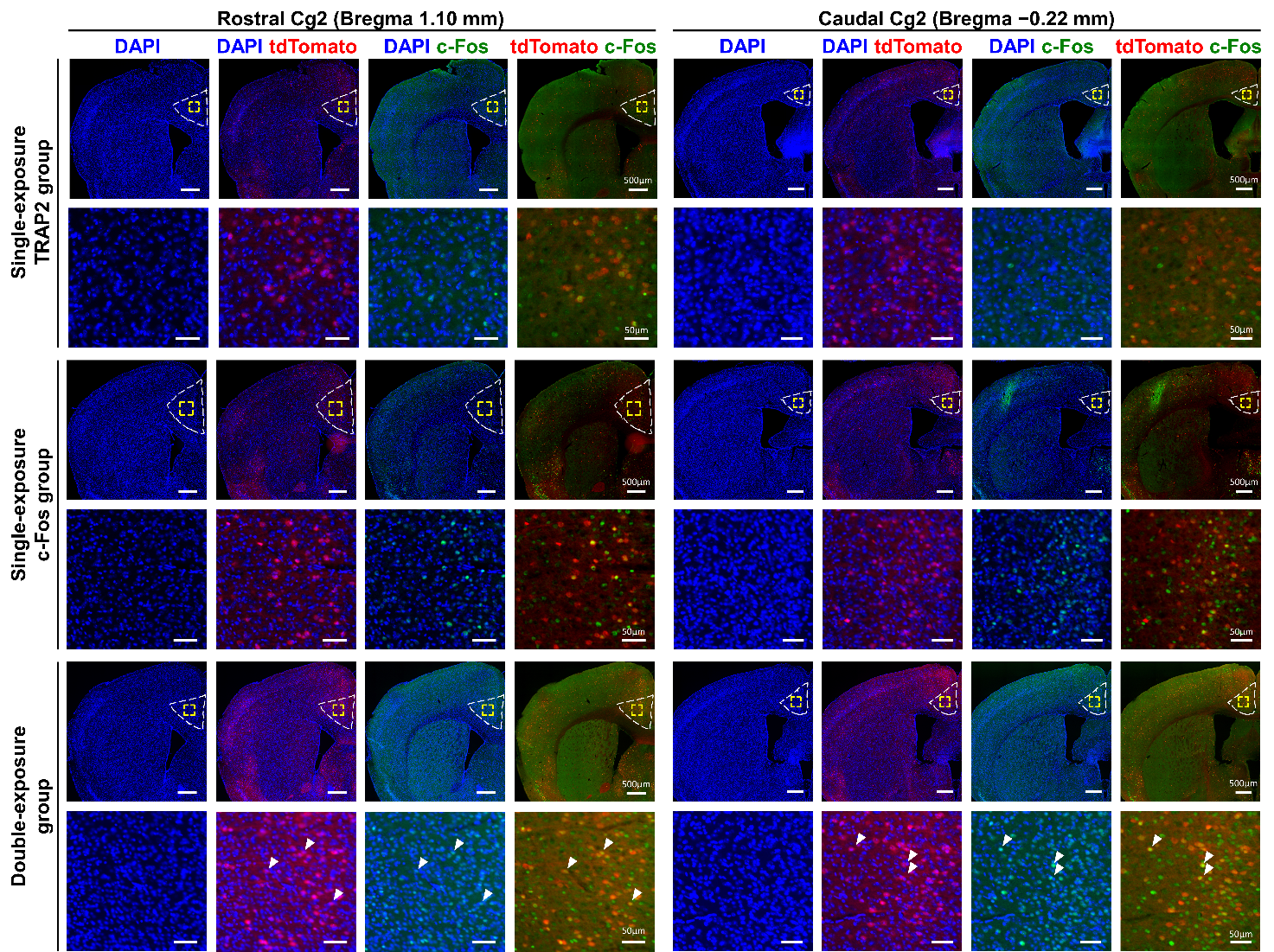
**

**
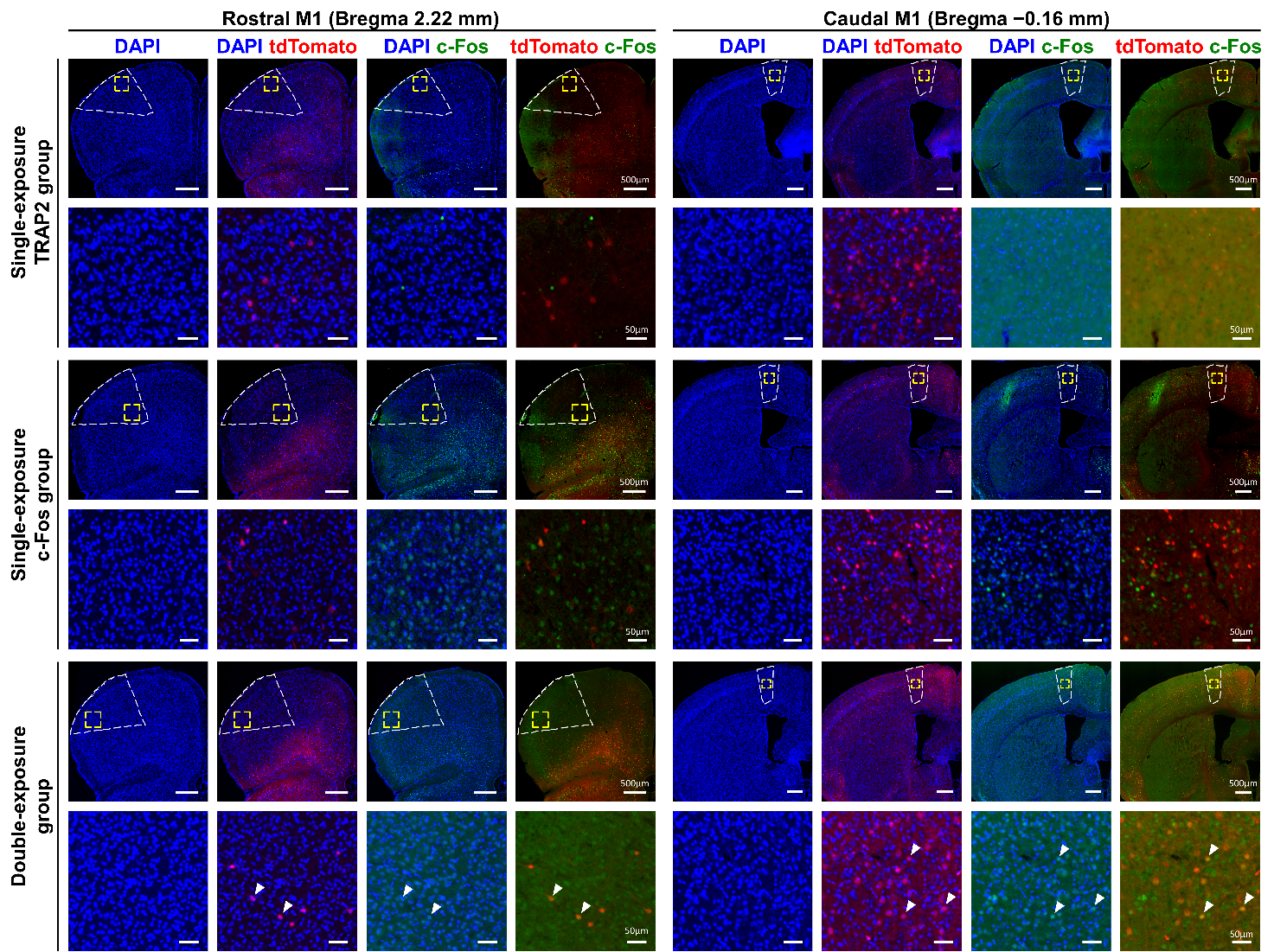
**

**
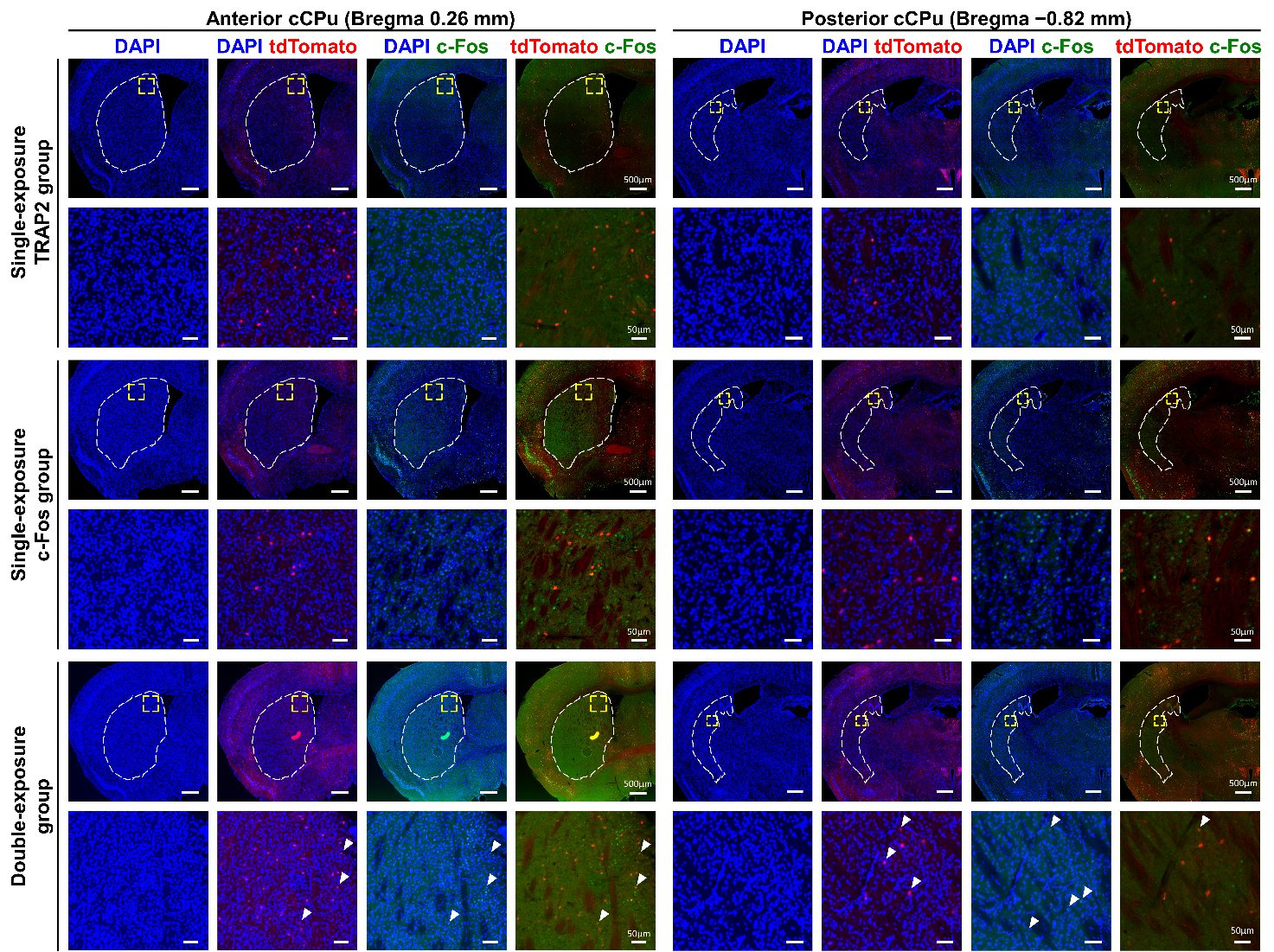
**

**
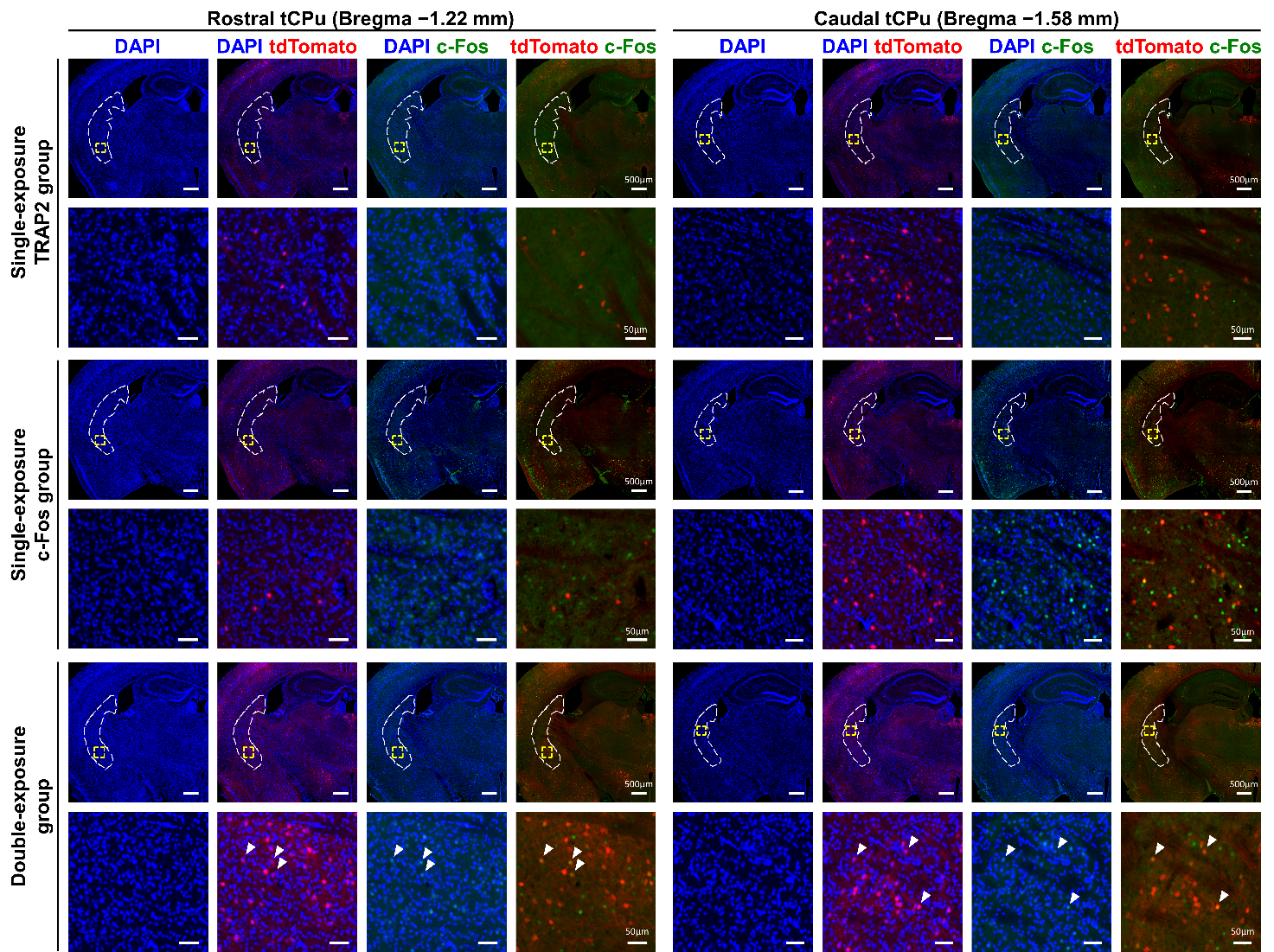
**

**
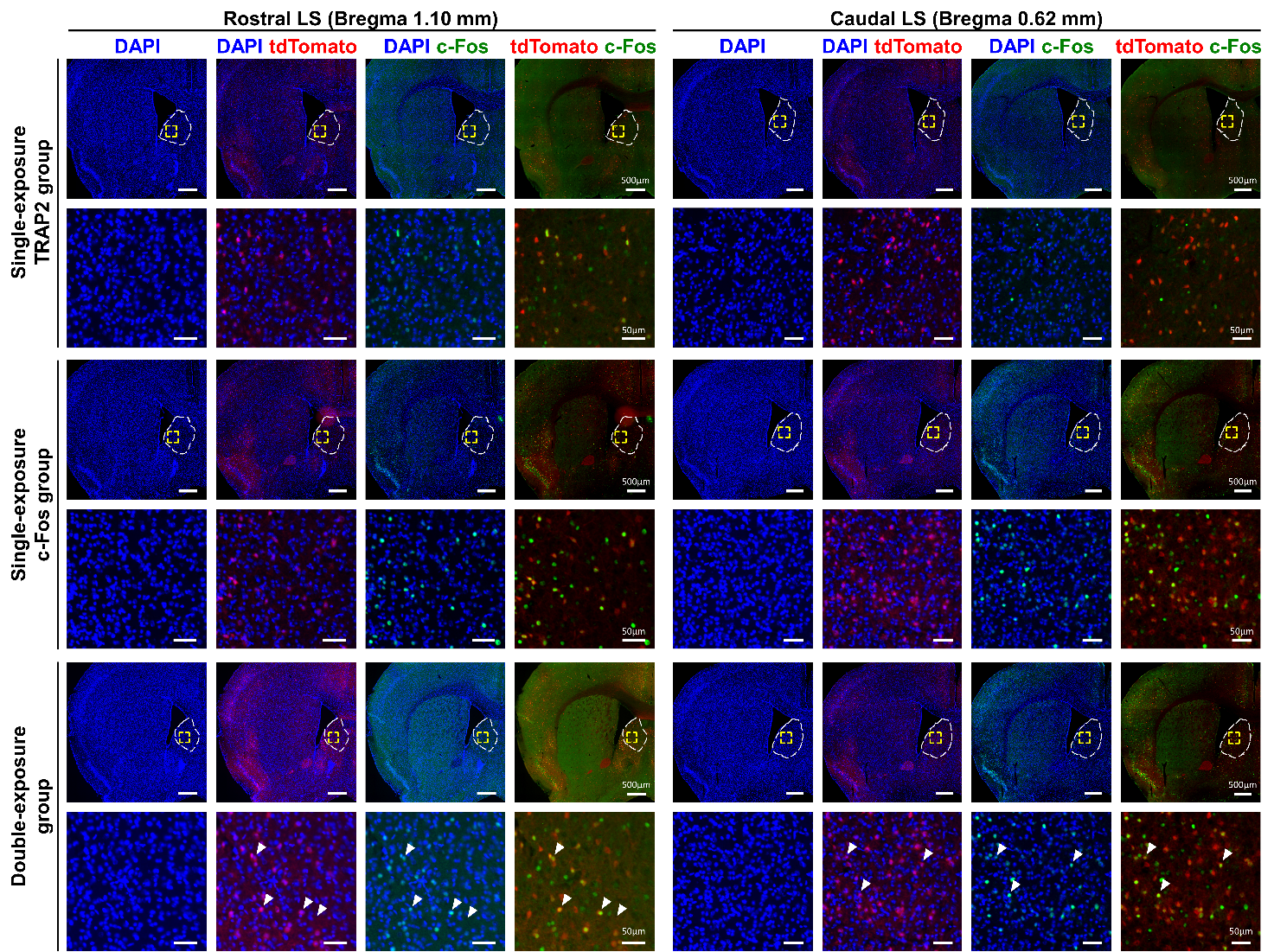
**

**
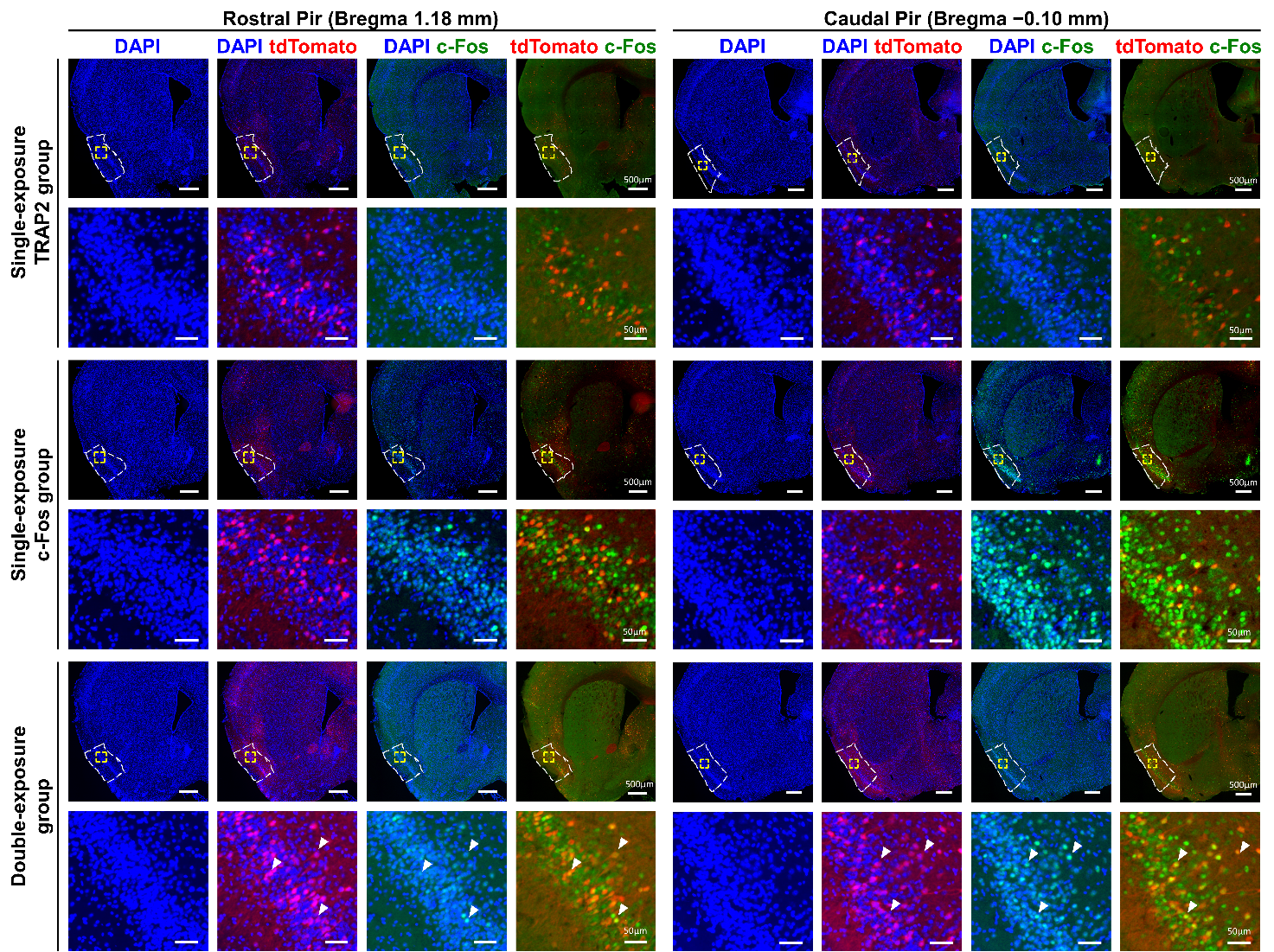
**

**
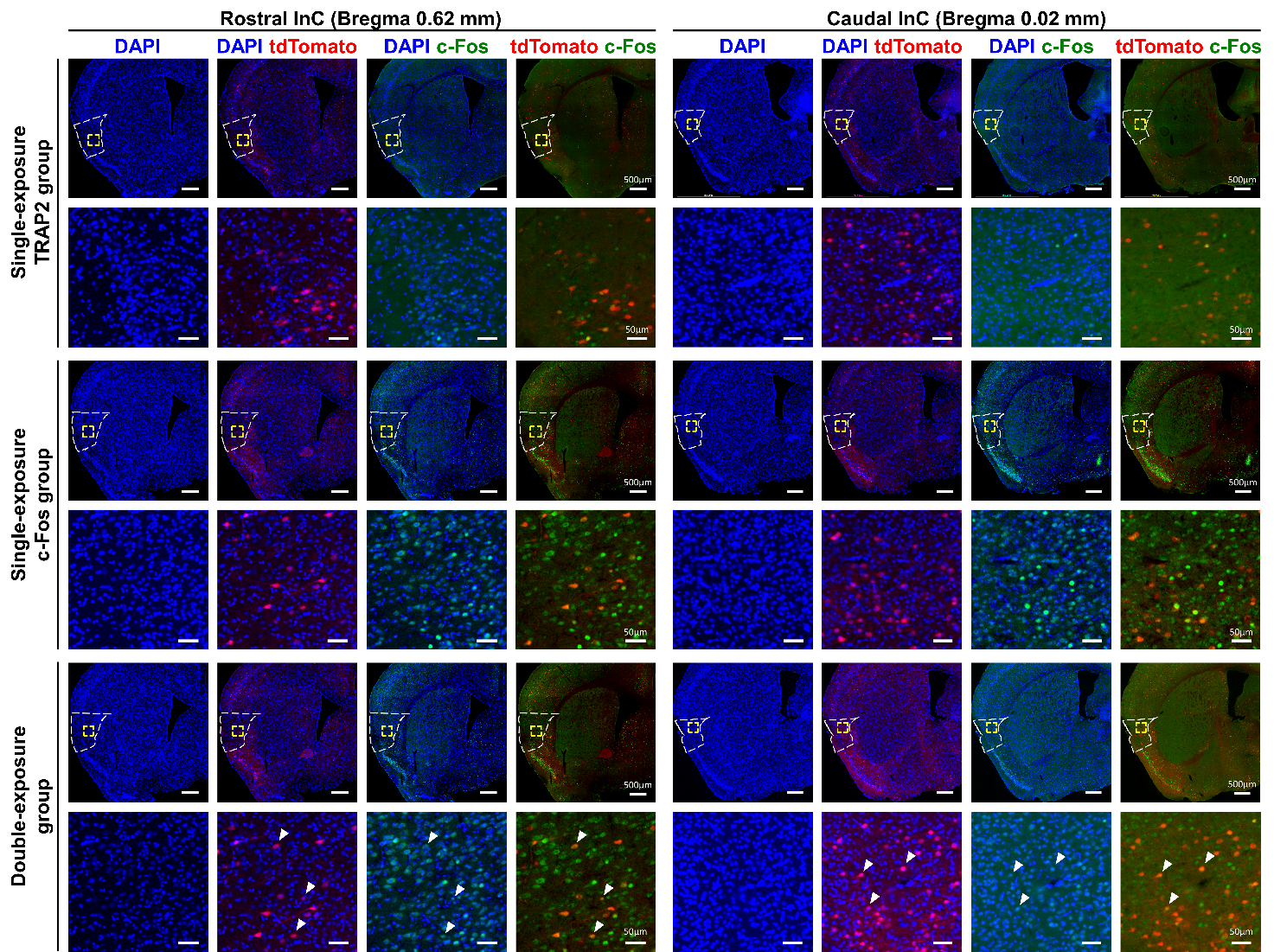
**

**
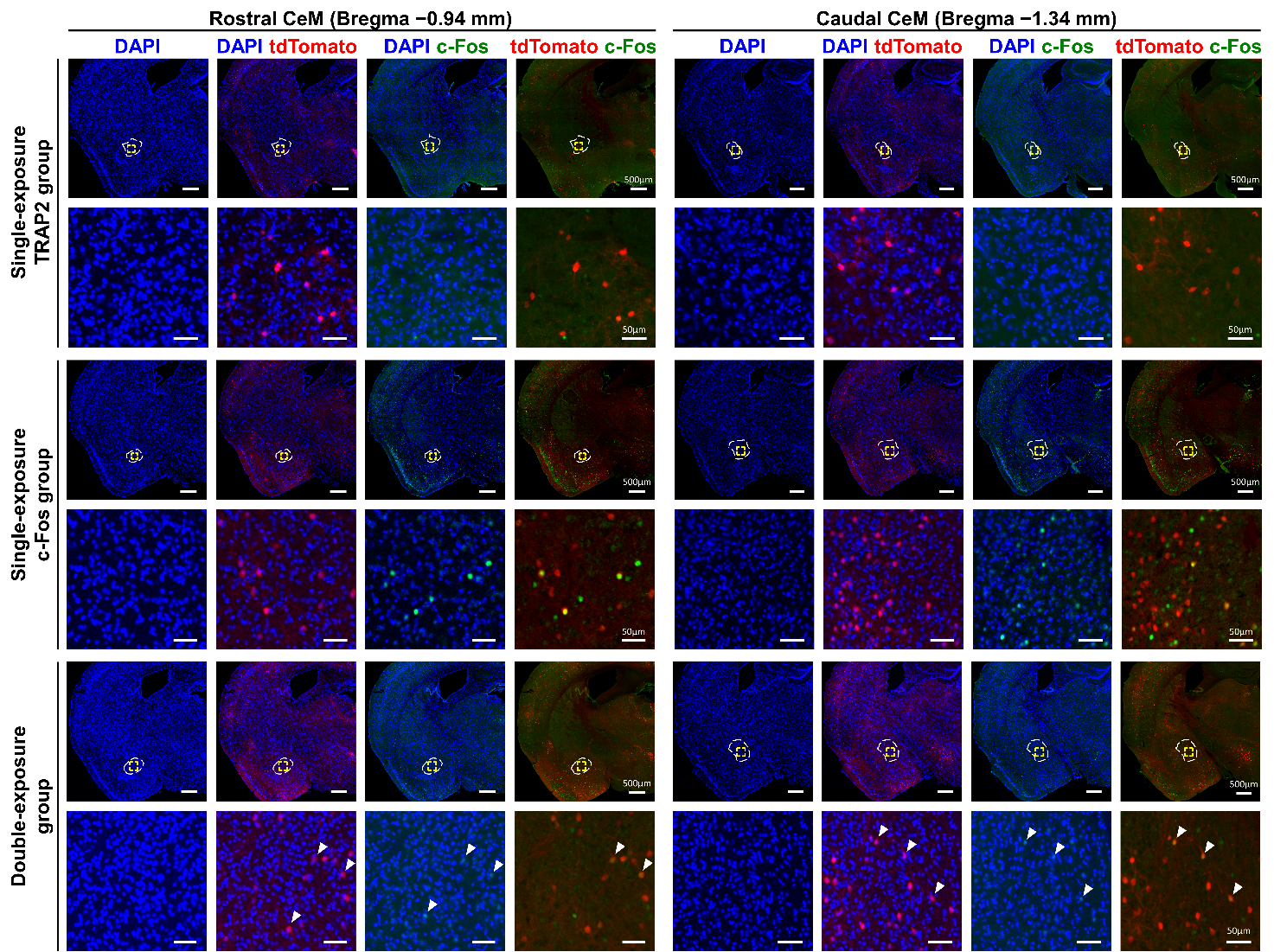
**

**
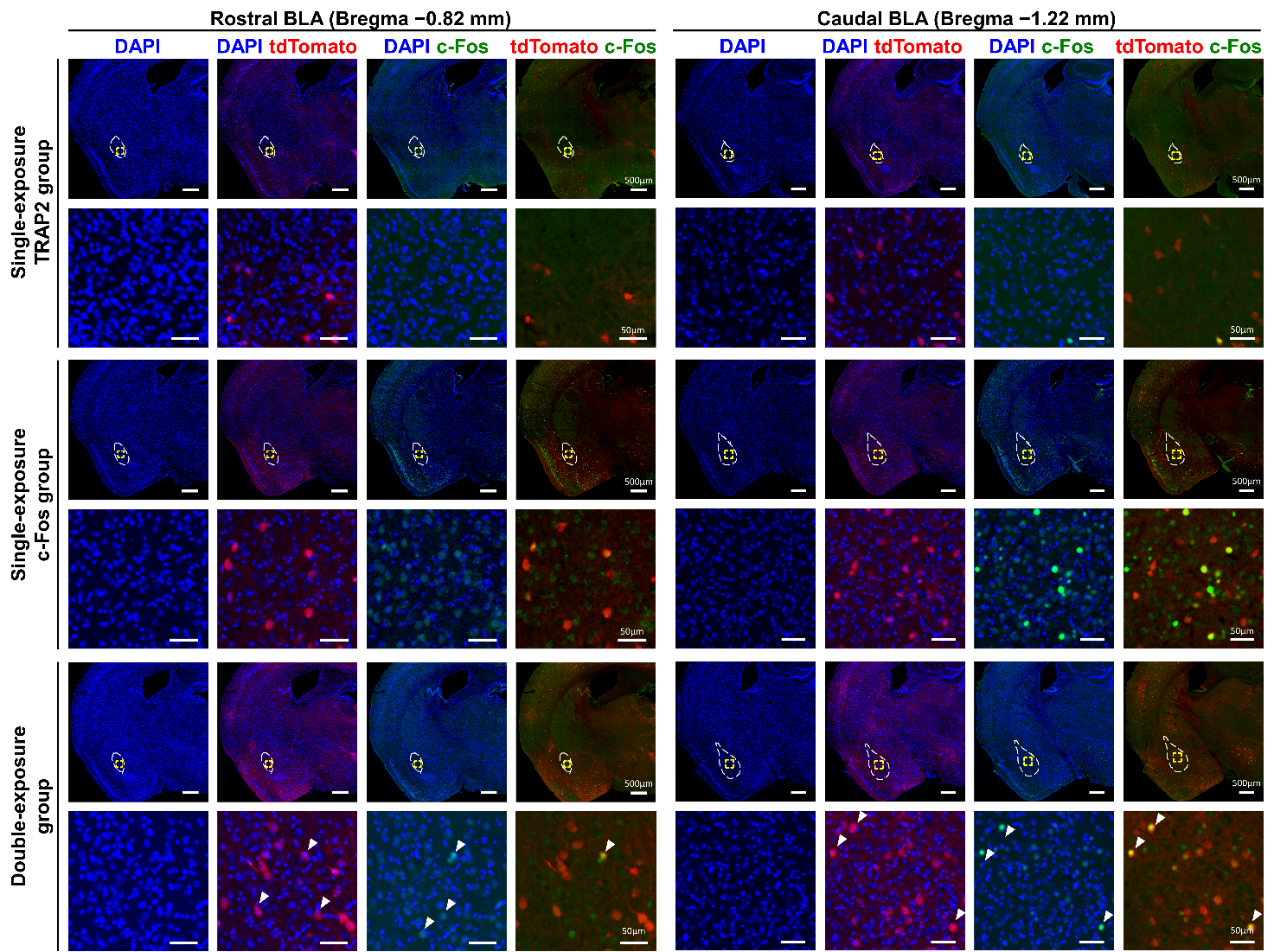
**

**
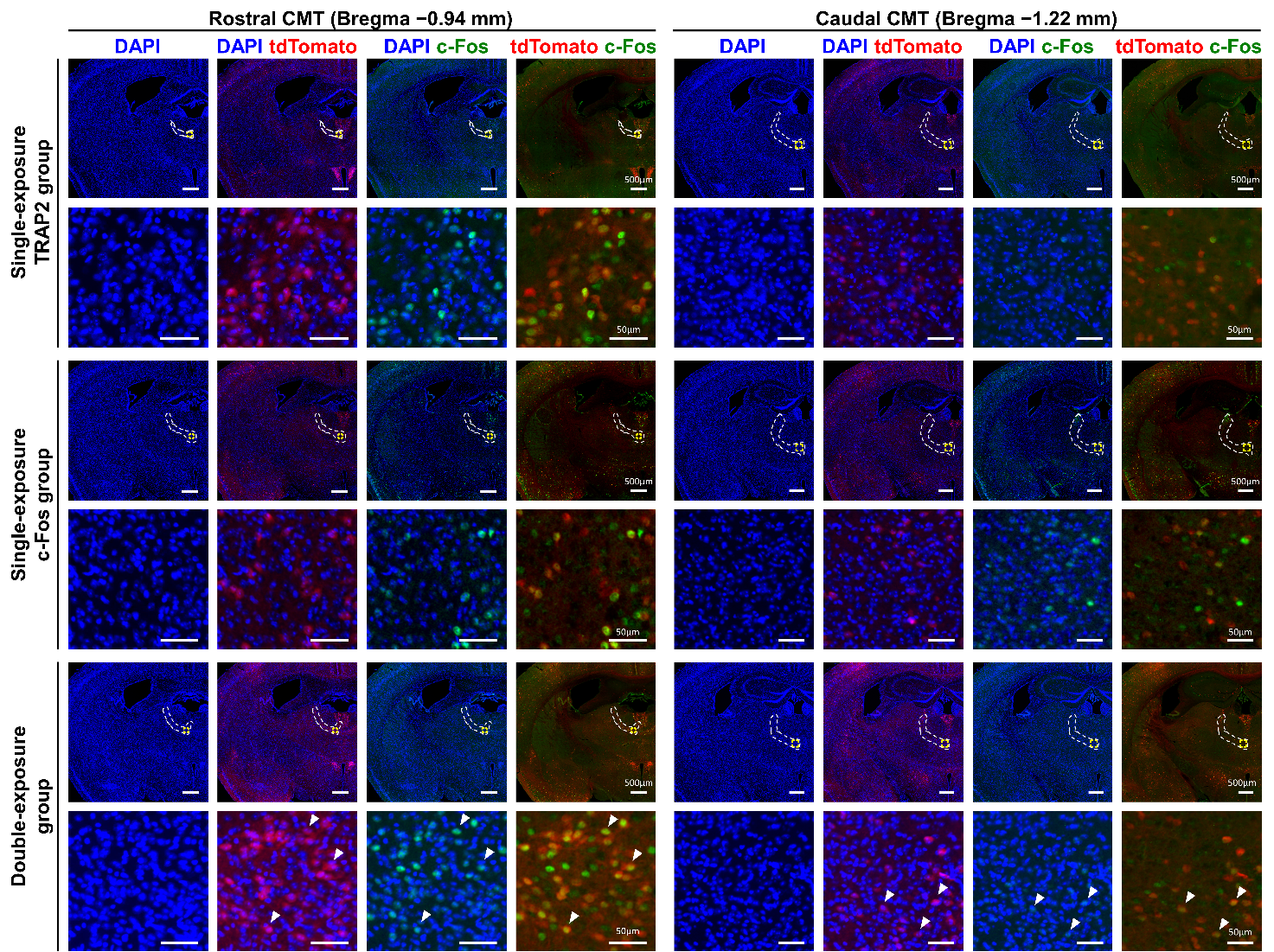
**

**
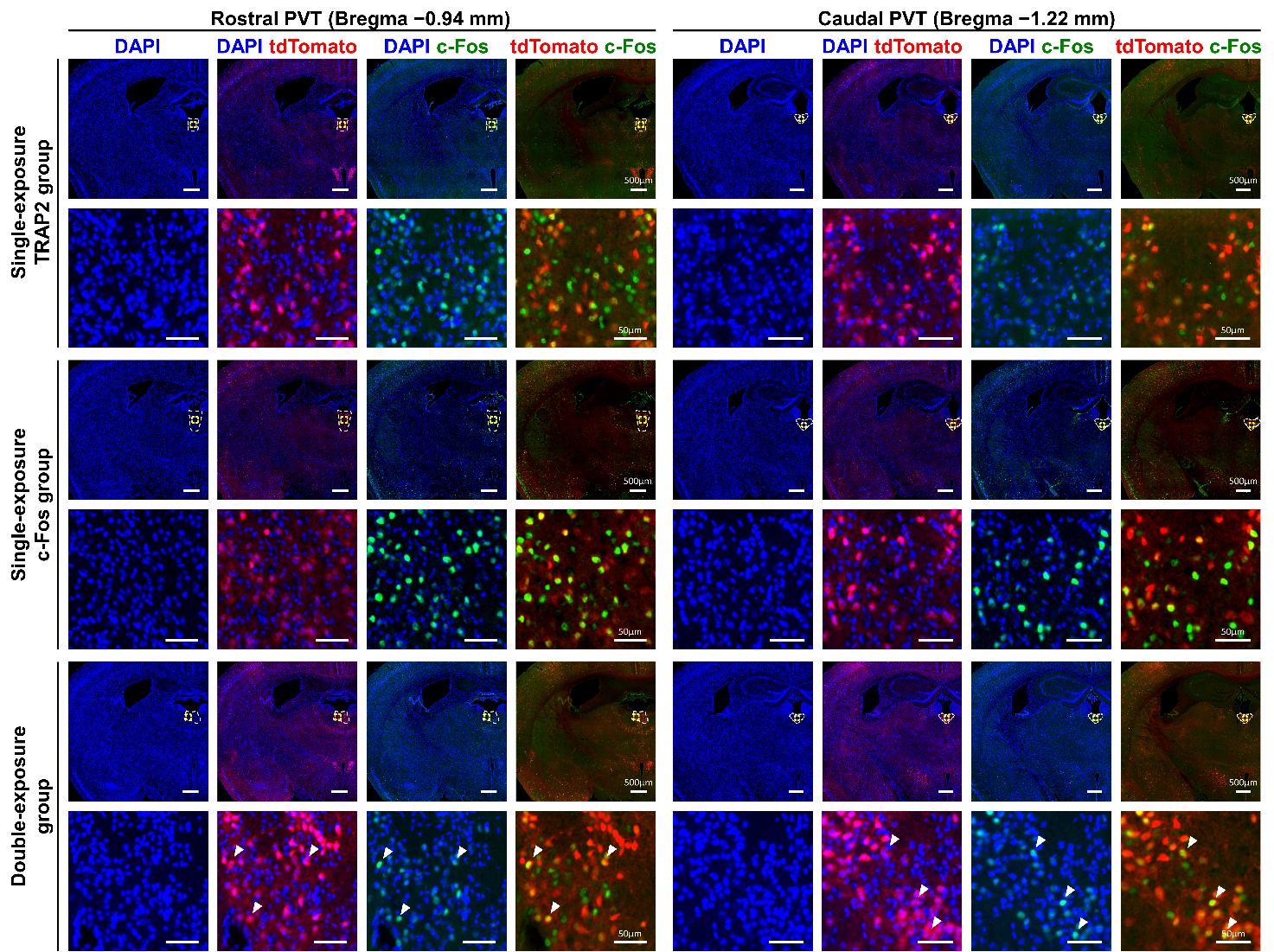
**

**
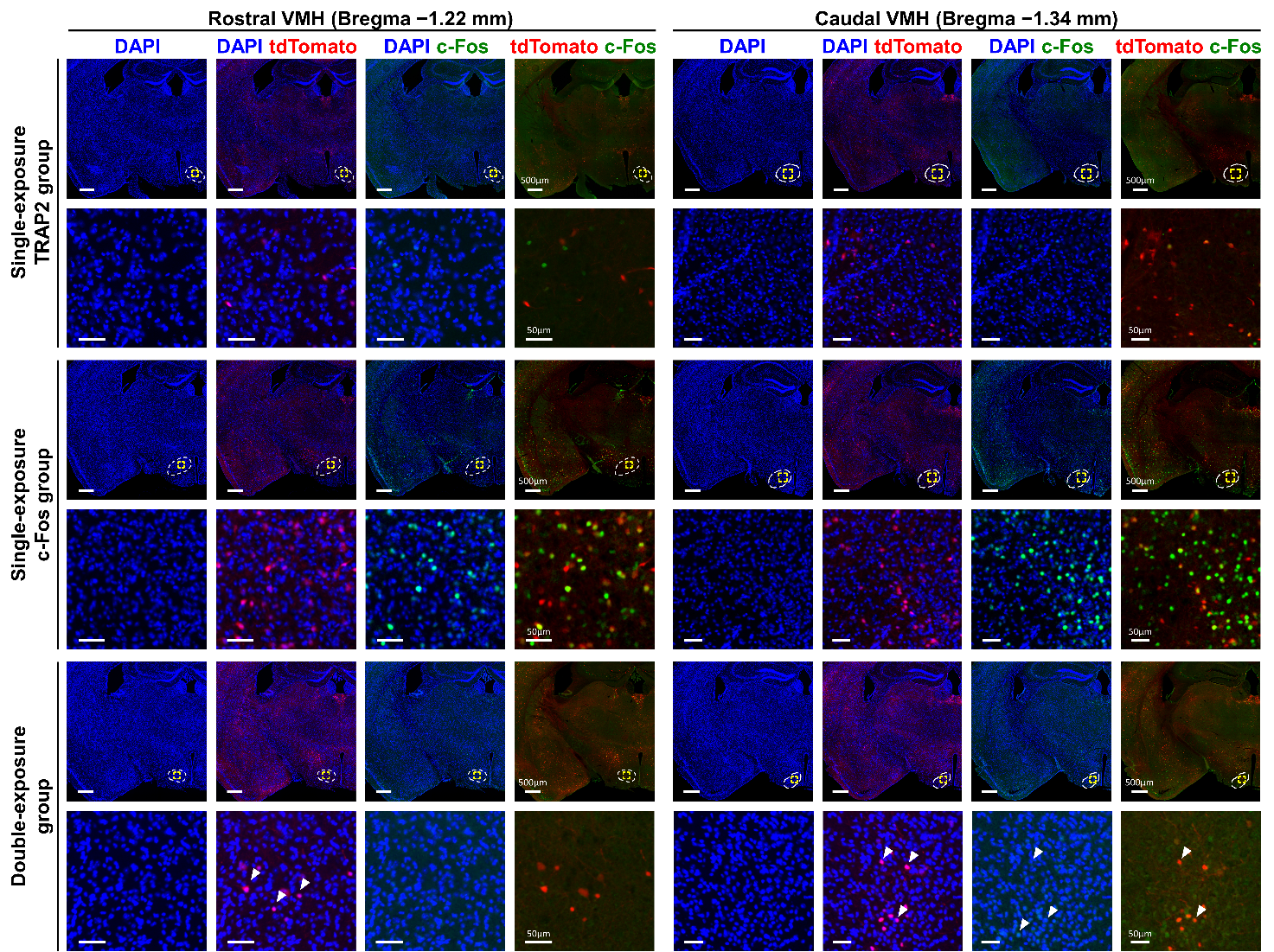
**

**
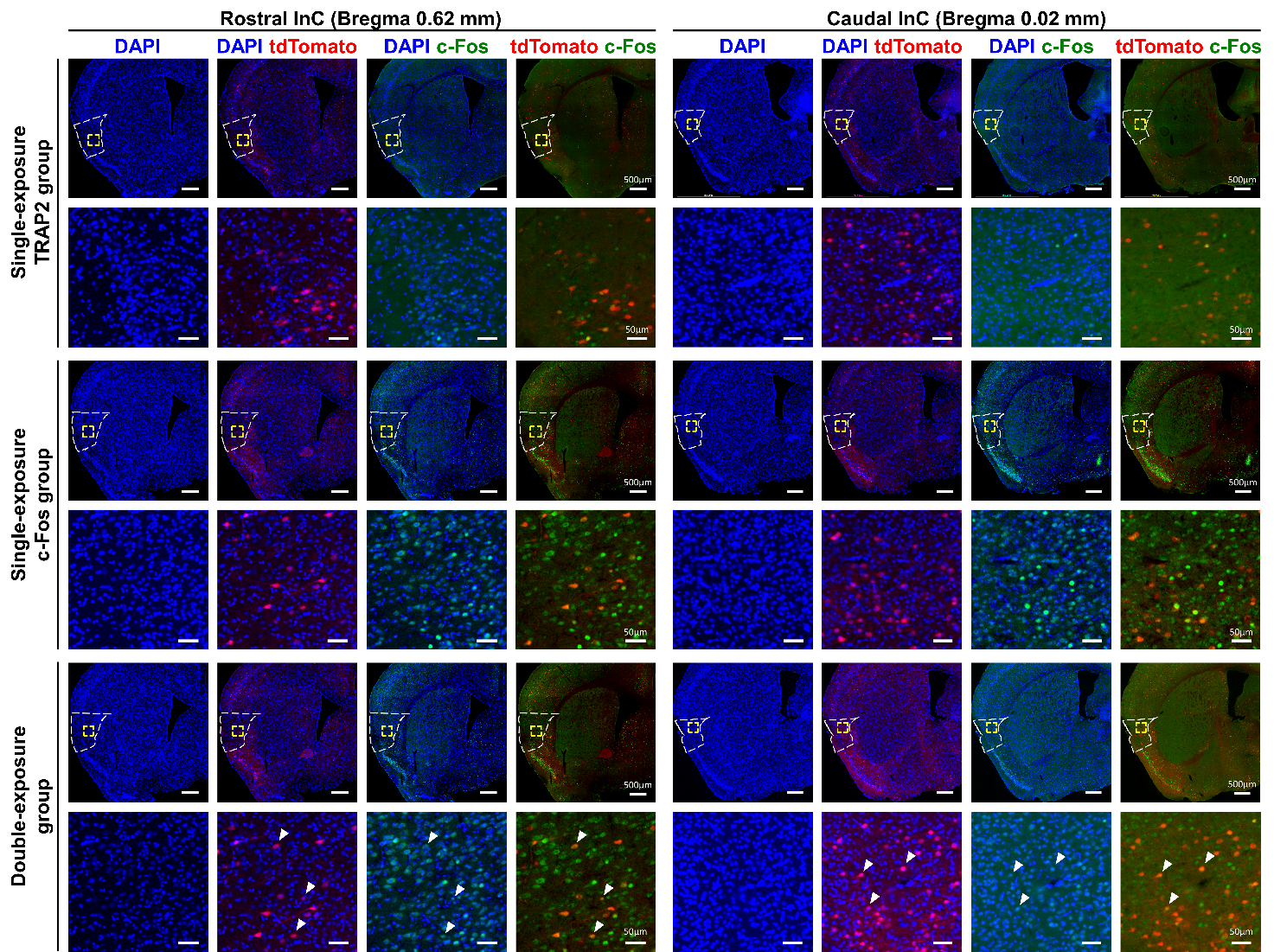
**

Supplement: Figure 2-1 — Download Figure 2-1, DOCX file. [file eneuro-13-ENEURO.0400-25.2026-s003.docx]
